# Calcium Ionophore (A23187) Rescues the Activation of Unfertilized Oocytes After Intracytoplasmic Sperm Injection and Chromosome Analysis of Blastocyst After Activation

**DOI:** 10.3389/fendo.2021.692082

**Published:** 2021-07-15

**Authors:** Ziwen Xu, Guidong Yao, Wenbin Niu, Huiying Fan, Xueshan Ma, Senlin Shi, Haixia Jin, Wenyan Song, Yingpu Sun

**Affiliations:** ^1^ Center for Reproductive Medicine, The First Affiliated Hospital of Zhengzhou University, Zhengzhou, China; ^2^ Henan Key Laboratory of Reproduction and Genetics, The First Affiliated Hospital of Zhengzhou University, Zhengzhou, China

**Keywords:** calcium ionophore (A23187), assisted reproductive technology (ART), intracytoplasmic sperm injection (ICSI), fertilization failure, artificial oocyte activation (AOA)

## Abstract

Calcium is a crucial factor in regulating the biological behavior of cells. The imbalance of calcium homeostasis in cytoplasm will cause abnormal behavior of cells and the occurrence of diseases. In intracytoplasmic sperm injection (ICSI) cycle, the dysfunction of oocyte activation caused by insufficient release of Ca^2+^ from endoplasmic reticulum is one of the main reasons for repeated fertilization failure. Calcium ionophore (A23187) is a highly selective calcium ionophore, which can form stable complex with Ca^2+^ and pass through the cell membrane at will, effectively increasing intracellular Ca^2+^ levels. It has been reported that calcium ionophore (A23187) can activate oocytes and obtain normal embryos. However, there are few studies on unfertilized oocytes after calcium ionophore (A23187) rescue activation in ICSI cycle. The purpose of this study was to analyze the effects of calcium ionophore (A23187) rescue activation on the activation of unfertilized oocytes, embryonic development potential, embryonic development timing and chromosomal aneuploidy, and to compare and analyze the clinical data of patients with calcium ionophore (A23187) activation in clinical application. The results showed that a certain proportion of high-quality blastocysts with normal karyotype could be obtained after calcium ionophore (A23187) rescue activation of unfertilized oocytes, and it did not have a significant effect on the timing of embryo development. In clinical practice, direct activation with calcium ionophore (A23187) after ICSI was better than rescue activation the next day. In conclusions, the studies on the effectiveness and safety of calcium ionophore (A23187) rescue activation for oocytes with ICSI fertilization failure can enable some patients to obtain usable, high-quality embryos during the first ICSI cycle.

## Introduction

The emergence of assisted reproductive technology (ART) has solved the problems of fertility and genetic diseases and improved the success rate of treatment for infertile couples worldwide. The application of intracytoplasmic sperm injection (ICSI) enables more couples who are infertile due to male factors, such as obstructive azoospermia and severe oligospermia or fertilization failure of previous *in vitro* fertilization (IVF) cycle, to achieve pregnancy (1). The ICSI technique, which uses a micromanipulator to inject a single sperm directly into the oocyte cytoplasm to fertilize oocytes, effectively improves the fertilization rate of single sperm, and the normal fertilization rate can reach more than 70%. Nevertheless, repeated fertilization failure or low fertilization still occurs in about 1%−5% of ICSI cycles clinically (2), which may be caused by the premature condensation of sperm chromatin, oocyte activation failure, failure to decondense sperm nuclei by oocytes, pronuclei formation disorder, et al. (3). Among these, oocyte activation failure is one of the main reasons for the failed fertilization after ICSI. In general, the activation process of oocytes is actually the transition process from the resumption of the second meiosis to the beginning of mitosis (4). During the normal fertilization process, the activation is triggered after the sperm head fuses with the oocyte membrane, increasing the level of inositol 1,4,5-trisphosphate (IP3) in the oocyte plasma. Then, IP3 binds to its receptor on the endoplasmic reticulum (ER), leading to Ca^2+^ release from ER. Finally, Ca^2+^ in the cytoplasm is reabsorbed into the ER. The level of Ca^2+^ in the oocyte plasma increases temporarily after this repeated process, which causes Ca^2+^ oscillations to reduce the activities of the metaphase promoting factor (MPF) and cytostatic factor (CSF), and then the oocyte restores meiotic ability. The continuous increase in Ca^2+^ level in the oocyte plasma to the formation of pronuclei is an important event for normal fertilization and embryonic development (5).

At present, several methods are available to activate the mammalian oocytes through physical and chemical stimulation, including electromechanical stimulation (6), calcium activation [calcium ionophore (A23187) or ionomycin], strontium chloride activation (7), phospholipase C zeta (PLCζ) activation (8), et al. The use of calcium ionophore (A23187) is one of the most efficient oocyte activation methods widely used in clinical practice. Calcium ionophore (A23187) has certain lipophilicity and can neutralize the positive charge of Ca^2+^. It can pump extracellular Ca^2+^ against the concentration gradient into the oocyte, increasing the concentration of Ca^2+^ in the oocyte plasma and ultimately activating the oocyte (9). For patients with repeated fertilization failures, the application of calcium ionophore (A23187) immediately after ICSI can significantly improve the fertilization and cleavage rates of mature oocytes and deliver successfully after embryo transfer (10–12).

It is difficult to diagnose and prevent fertilization failure in advance after ICSI treatment. Therefore, it is important to find ways to improve the fertilization rate and clinical outcome (13). In general, when fertilization failure occurs in the first ICSI cycle, artificial oocyte activation (AOA) is used in subsequent cycles to prevent the reoccurrence of fertilization failure. However, if oocytes with fertilization failure are activated and continue to develop with calcium ionophore (A23187) after 18-24 h in the first ICSI cycle, the patient will obtain available embryos in this cycle.

In this study, the embryonic development of patients with the activation of calcium ionophore (A23187) immediately after the ICSI due to fertilization failure in the last cycle was analyzed and compared with the development in those with complete fertilization failure after ICSI who received rescue activation of calcium ionophore (A23187) on the following day. Moreover, 101 clinically discarded oocytes with fertilization failure 18–24 h post-ICSI were collected, and AOA was performed with calcium ionophore (A23187) to observe the fertilization rate, potential of embryo development, assessment of dynamic embryo development, and Single Nucleotide Polymorphism (SNP) analysis of the obtained blastocysts.

## Materials and Methods

### Sample Collection

In this study, the clinical data of 58 patients who underwent ICSI fertilization and calcium ionophore (A23187) activation from April 2018 to February 2020 in the Center for Reproductive Medicine of the First Affiliated Hospital of Zhengzhou University were retrospectively analyzed and divided into three groups. In the RA group (calcium ionophore (A23187) rescue activation group), 77 MII oocytes were activated with calcium ionophore (A23187) in 7 patients who did not show pronuclei 18-24 h post-ICSI. In the DA group (calcium ionophore (A23187) direct activation after ICSI), 525 MII oocytes were collected in 43 patients who were activated by calcium ionophore (A23187) immediately after ICSI in this cycle due to previous fertilization failure or poor fertilization of ICSI. In the DA-PGT group [calcium ionophore (A23187) direct activation after ICSI, followed by preimplantation genetic testing (PGT)], 43 MII oocytes were activated with calcium ionophore (A23187) immediately after ICSI in 8 patients who had fertilization failure or poor fertilization in the previous ICSI cycle, followed by PGT on the trophoblast cells from the formed blastocysts.

Total of 374 metaphase II (MII) oocytes from 22 patients undergoing ICSI treatment were collected from April 2018 to April 2019. In RA-C group (calcium ionophore (A23187) rescue activation treatment control group), 374 MII oocytes were collected after routine ICSI from 22 patients; In RA-T group (calcium ionophore (A23187) rescue activation treatment experimental group), the 101 unfertilized MII oocytes from RA-C group without pronuclei and no signs of fertilization 18-24 h post-ICSI were collected for calcium ionophore (A23187) rescue activation.

This study was approved by the Ethics Committee of the First Affiliated Hospital of Zhengzhou University, and all included patients signed informed consent.

### Materials and Methods

#### Intracytoplasmic Sperm Injection

The cumulus oocyte complexes (COCs) were retrieved after 36-h hCG injection under the guidance of transvaginal B-ultrasound. The retrieved COCs were immediately put into G-IVF Plus (Vitrolife Sweden AB, Goteborg, Sweden) medium and cultured for 1-2 h at 37°C and in the presence of 6% CO_2_. Spermgrad (Vitrolife) at concentrations of 90% and 45% was used for density gradient centrifugation and G-IVF Plus for washing the ejaculated sperm. The testicular tissues from patients undergoing testicular sperm aspiration were shredded in G-IVF Plus solution with a 1-mL syringe needle, centrifuged, and washed directly. The granulosa cells around COCs were removed by repetitive aspiration with a 150-μm-diameter fine needle (Sunlight, FL, USA) with hyaluronidase (Vitrolife) digestion. Then, the oocytes removed from the granulosa cells were cultured in G-IVF Plus for 1–2 h for ICSI. After injection, the oocytes were cultured in a 50-μL G-1 Plus (Vitrolife) droplet covered with paraffin oil (Vitrolife) and cultured at 37°C and in the presence of 6% CO_2_.

#### Calcium Ionophore (A23187) Activation

The oocytes were activated with an incubator containing 10 μM calcium ionophore (A23187) (Sigma, MO, USA) prepared using G-1 Plus at 37°C and in the presence of 6% CO_2_ for 10 min after ICSI. The oocytes after activation were washed three times with G-1 Plus and then transferred to G-1 Plus droplets for subsequent culture.

#### Pronuclear Observation and Embryo Culture

The fertilization of oocytes was observed under an inverted microscope (TE2000-U, Nikon, Japan) at 16-18 h post-ICSI. However, due to the difference in the developmental ability of oocytes, we will observe again between 18-24 h to confirm the fertilization status of the oocytes. By observing the pronuclei formation and polar body extrusion, the successful fertilization after the activation of calcium ionophore (A23187) were determined: one pronuclei with second polar body extrusion (1PN + 2PB), two pronuclei with first polar body extrusion (2PN + 1PB), two pronuclei with second polar body extrusion (2PN + 2PB), the presence of three or more pronuclei (≥3PN), and direct cleavage (14). The total fertilization rate = the number of oocytes with one or more PN/the total number of MII oocytes activated × 100%, the abnormal fertilization rate =the number of abnormal fertilized oocytes/the total number of MII oocytes activated ×100%, and the normal fertilization rate = the number of normal fertilized oocytes/the total number of MII oocytes activated × 100%

The oocytes were cultured in G-1 Plus droplets to observe their developmental potential after calcium ionophore (A23187) activation. The embryo grade was evaluated on the Day3 (D3) of embryo development according to the Peter scoring system (15). Embryos with more than six blastomeres and less than 10% fragments were evaluated as high-quality embryos. Early cleavage rate = the number of cleaved embryos derived from normal fertilization 26-27 h after activation/the number of normal fertilized oocytes × 100%, Day2 (D2) cleavage rate = the number of cleaved embryos derived from normal fertilization 44-46 h after activation/the number of normal fertilized oocytes × 100%, D3 embryo formation rate = the total number of formed embryos/the number of total cleaved embryos derived from normal fertilization× 100%, D3 high-quality embryo rate = the number of D3 high-quality embryos/the number of total cleaved embryos derived from normal fertilization× 100%.

#### Blastocyst Culture and Biopsy

The embryos were transferred to a 50μL G-2 Plus (Vitrolife) droplet covered with paraffin oil for further culture until Day5 (D5) or Day6 (D6). The blastocysts were observed under an inverted microscope and scored according to the Gardner blastocyst scoring system (16). The blastocysts with grades equal to or higher than 3BB on D5 and those with grades equal to or higher than 4BB on D6 were evaluated as high quality. Blastocyst formation rate = the total number of formed blastocysts/the number of embryos for blastocyst culture×100%, high-quality blastocyst rate = the total number of high-quality blastocysts/the number of embryos for blastocyst culture×100%.

In the afternoon of the fourth day of embryonic development, a laser-assisted hatching system (ZILOS-tk, Hamilton Thorne Biosciences, Beverly, MA, USA) was used to make a small hole in the zona pellucida of the embryo away from inner cell mass (ICM) to facilitate the hatching of trophoblast cells (TE). For blastocysts with prominent blastocyst cavity on D5/6, three to five TE cells were taken out with a biopsy needle (Origio, VA, USA) with an inner diameter of 25μm for chromosome aneuploidy analysis under an inverted microscope (Nikon) using a micro-operation system (Narashige, Japan).

#### Chromosome Aneuploidy Analysis

The TE cells in blastocysts from the RA-T group were analyzed for chromosome aneuploidy using SNP microarray analysis, as described previously (17). The next-generation sequencing was used for analyzing clinical PGT chromosome aneuploidy, which was described in a previous study (17).

### Statistical Analysis

The activation rate, cleavage rate, and other data in the two groups were compared using SPSS (version 23, IBM corp., Armonk, NY, USA) and GraphPad Prism 7.0 (GraphPad Software Inc., San Diego, CA, USA) software. The quantitative data were expressed as mean ± standard deviation (χ2 ± SD), and the differences were compared using the *t-*test and *F*-test. The qualitative data were expressed as *n* (%). The binary variables in the two groups were compared using the chi-square test, and the rank variables were compared using the rank-sum test. *P* < 0.05 indicates a significant difference.

## Results

### Comparison of General Characteristics of Patients in Different Groups

A total of 80 patients were selected in this study, including 22 patients in the RA-T and RA-C groups, 7 patients in the RA group of rescue clinical activation, 43 patients in the DA group of direct activation after ICSI, and 8 patients in the DA-PGT group of direct activation after ICSI and PGT treatment. The results showed no significant differences in age, body mass index (BMI), basal follicle stimulating hormone (FSH) levels, and estradiol (E_2_) levels among the groups (*P* > 0.05), but significant differences were observed in basal luteinizing hormone (LH) and anti-mullerian hormone (AMH) levels between the RA group and other groups (*P* < 0.05) (**Table 1**).

**Table 1:** Comparisons of general characteristics of patients in each group.

	RA Group	DA Group	DA-PGT Group	RA-T/C Group
No. of cases	7	43	8	22
Age (year)	31.4 ± 5.2	29.4 ± 4.6	29.8 ± 1.8	28.6 ± 4.0
BMI (kg/m^2^)	22.0 ± 2.1	23.5 ± 3.3	21.9 ± 4.0	24.1 ± 2.9
FSH (mIU/ml)	5.8 ± 1.3	7.2 ± 2.7	6.9 ± 1.3	6.2 ± 1.4
LH (mIU/ml)	2.2 ± 1.2*	5.4 ± 3.3	5.6 ± 1.3	5.5 ± 3.0
E_2_ (pg/ml)	32.5 ± 17.0	35.1 ± 18.3	40.9 ± 13.2	40.4 ± 19.8
AMH (ng/ml)	2.4 ± 1.3*	3.6 ± 2.8	3.4 ± 1.3	4.8 ± 2.9

The data were expressed as mean ± SD. RA indicates that the oocytes were activated with calcium ionophore (A23187) in 7 patients who did not show pronuclei 18-24 h after ICSI in the first cycle. DA represents that the MII oocytes were collected in 43 patients who were activated by calcium ionophore (A23187) immediately after ICSI in this cycle due to previous fertilization failure or poor fertilization of ICSI. DA-PGT refers to patients who were activated by calcium ionophore (A23187) immediately after ICSI due to fertilization failure or poor fertilization in the previous ICSI cycle, followed by PGT on the trophoblast cells from the formed blastocysts. RA-C indicates that the MII oocytes were collected after routine ICSI from 22 patients; RA-T represents that the unfertilized MII oocytes from RA-C group without pronuclei and no signs of fertilization 18-24 h post-ICSI were collected for calcium ionophore (A23187) rescue activation. BMI, body mass index; FSH, follicle stimulating hormone; LH, luteinizing hormone; E_2_, estradiol; AMH, anti-mullerian hormone. ^*^P < 0.05.

### Effects of Calcium Ionophore (A23187) Rescue Activation and Direct Activation After ICSI on Embryo Development

Oocytes cultured *in vitro* for a long time led to oocyte aging, affecting oocyte fertilization and embryonic development. Therefore, whether the rescue activation of calcium ionophore (A23187) affects the fertilization and embryonic development of oocytes without pronuclei and no signs of fertilization 18-24 h post-ICSI needed exploration. This study retrospectively analyzed patients who underwent calcium ionophore (A23187) rescue activation due to complete fertilization failure after ICSI (RA group) and patients who underwent calcium ionophore (A23187) activation directly after this cycle of ICSI (DA group) due to poor fertilization or complete fertilization failure in the last cycle. A total of 77 MII oocytes from 7 patients in the RA group and 525 MII oocytes from 43 patients in the DA group were included. The fertilization, cleavage, and embryo development were analyzed and compared between the two groups (**Table 2**). The results showed that the total fertilization rate, abnormal fertilization rate, normal fertilization rate and D3 embryo formation rate in the RA group were not significantly different from those in the DA group (*P* > 0.05). But the early cleavage rate, D2 cleavage rate, D3 high-quality embryo rate, blastocyst formation rate, and high-quality blastocyst rate in the DA group were significantly higher than those in the RA group (*P* < 0.05). These results indicated that the developmental potential of embryos directly activated using calcium ionophore (A23187) after ICSI was superior to rescue activated embryos with no pronuclei or signs of fertilization on the next day after ICSI.

**Table 2:** Comparison of the effects of calcium ionophore (A23187) rescue activation and direct activation on embryonic developmental potential after ICSI.

	RA Group	DA Group	*P*-value
No. MII oocytes	77	525	–
Total fertilization rate (%)	67.5 (52/77)	58.3 (306/525)	0.123
Abnormal fertilization rate (%)	5.2 (4/77)	3.1 (16/525)	0.326
Normal fertilization rate (%)	62.3 (48/77)	55.2 (290/525)	0.241
Early cleavage rate (%)	4.2 (2/48)	37.9 (110/290)	0.000
D2 cleavage rate (%)	89.6 (43/48)	96.9 (281/290)	0.019
D3 embryo formation rate (%)	97.7 (42/43)	91.5 (257/281)	0.155
D3 high-quality embryo rate (%)	23.3 (10/43)	56.9 (160/281)	0.000
Blastocyst formation rate (%)	10.5 (4/38)	44.0 (74/168)	0.000
High-quality blastocyst rate (%)	0.00 (0/38)	14.3 (24/168)	0.000

The values in the table are expressed as percentages. D2, day2. D3, day3. P<0.05 indicates a significant difference.

In RA group, 4 high-quality embryos were frozen, and the remaining 6 high-quality embryos and 32 non-high-quality embryos continued to be cultured. Four non-high-quality blastocysts were obtained. Because the development of embryo derived from calcium ionophore (A23187) rescue activation is not synchronized with the development of endometrial, the embryos derived from the rescue activation are not freshly transferred in this cycle, and the formed blastocysts are frozen. Through further analysis of the results of freeze-thawing transfer in the RA group, it was found that 4 non-high-quality blastocysts were thawed and transferred without pregnancy. However, one patient who underwent embryo freeze-thaw transfer received a live birth last year and is currently in normal health and intelligence. In the DA group, 37 high-quality embryos and 2 high-quality blastocyst were freshly transferred in this cycle, resulting in 18 live births. Nevertheless, the embryonic development quality of the rescue activation group is not as good as that of the direct activation group, which may be related to some problems in these embryos.

### Effect of Calcium Ionophore (A23187) Rescue Activation Treatment on Oocyte Activation and Subsequent Embryo Development

According to the previously published reports and our pre-experimental research, a certain proportion of well-developed embryos and blastocysts were obtained by rescue activation with calcium ionophore (A23187) for oocytes without pronuclei and no signs of fertilization 18–24 h post-ICSI. Hence, the discarded oocytes with no pronuclei or signs of fertilization 18–24 h post-ICSI were collected and treated with calcium ionophore (A23187) for rescue activation (RA-T) and compared with the oocytes from patients with normal oocytes after ICSI (RA-C). The total fertilization rate, normal fertilization rate, early cleavage rate, D2 cleavage rate, D3 high-quality embryo rate, blastocyst formation rate, and high-quality blastocyst formation rate were compared between the two groups (**Table 3**). Compared with the RA-C group, no significant difference was found in early cleavage rate, blastocyst formation rate, and high-quality blastocyst formation rate between the RA-T and RA-C groups (*P* < 0.05). However, the rates of D2 cleavage rate, D3 embryo formation rate and D3 high-quality embryo rate in the RA-C group were significantly higher than those in the RA-T group (*P* = 0.000).

**Table 3:** Comparison of the effects of calcium ionophore (A23187) rescue activation and normal ICSI treatment on oocyte fertilization and subsequent embryonic development potential.

	RA-C Group	RA-T Group	*P* value
No. oocytes	374	101	–
Total fertilization rate (%)	68.7 (257/374)	76.2 (77/101)	–
Abnormal fertilization rate (%)	1.1 (4/374)	35.6 (36/101)	–
Normal fertilization rate (%)	67.6 (253/374)	40.6 (41/101)	–
Early cleavage rate (%)	39.5 (100/253)	26.8 (11/41)	0.813
D2 cleavage rate (%)	98.0 (248/253)	58.5 (24/41)	0.000
D3 embryo formation rate (%)	89.1 (221/248)	50.0 (12/24)	0.000
D3 high-quality embryo rate (%)	60.1 (149/248)	33.3 (8/24)	0.000
Blastocyst formation rate (%)	64.0 (112/175)	66.7 (8/12)	0.440
High-quality blastocyst rate (%)	21.1 (37/175)	33.3 (4/12)	0.323

The values in the table are expressed as percentages. P < 0.05 indicates a significant difference.

### Effect of Calcium Ionophore (A23187) Rescue Activation on Embryonic Development Timing After Oocyte Activation

**Figure 1:**
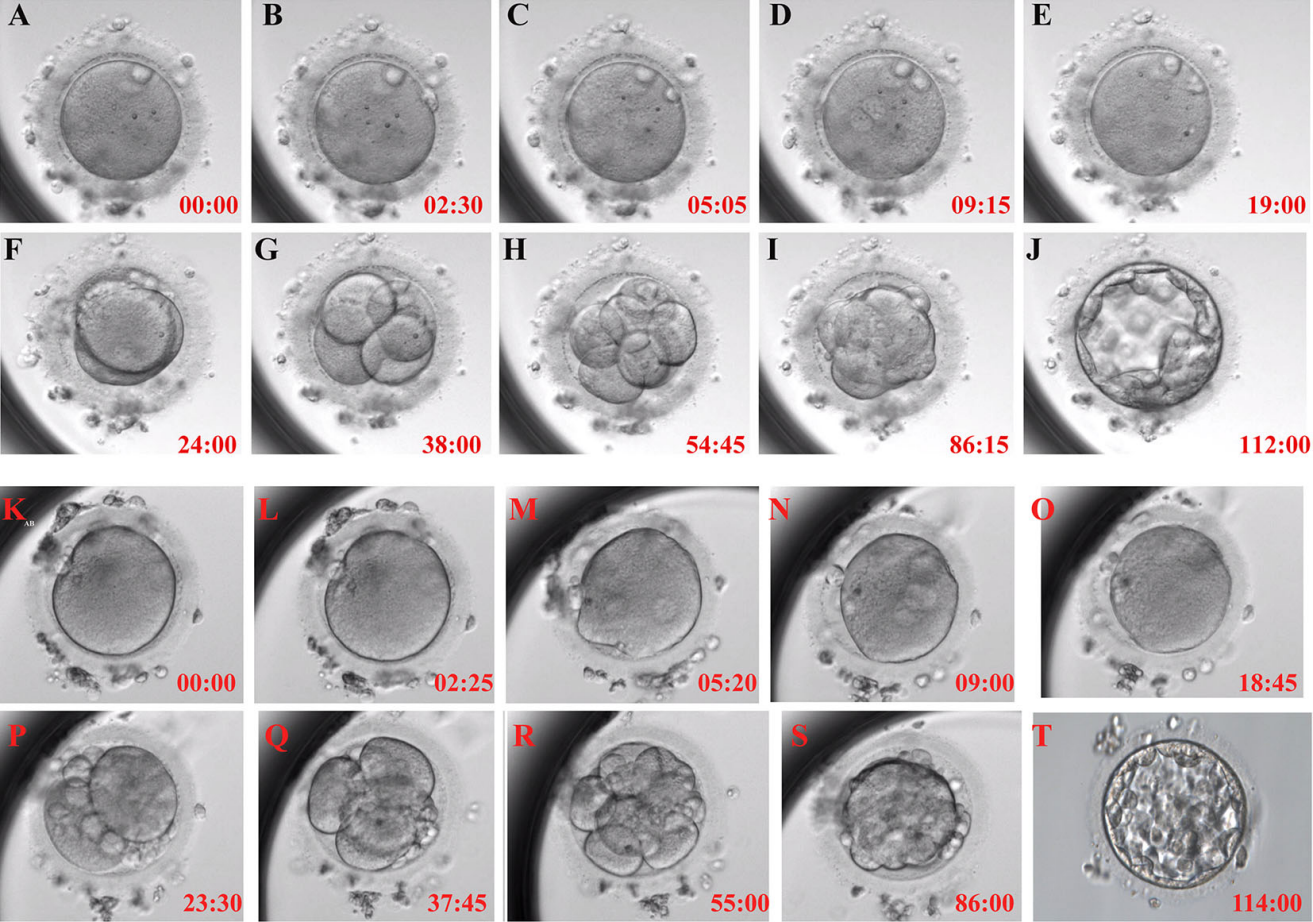
Typical embryonic development pattern of calcium ionophore (A23187) rescue activation. Each figure shows the activation process for 0h **(A)**, the second polar body appearance **(B)**, pronuclei appearance **(C)**, pronuclei volume is the largest and most obvious **(D)**, pronuclei disappear **(E)**, the first cleavage **(F)**, 4-cell stage **(G)**, 8-cell stage **(H)**, morula formation **(I)** and blastocyst formation **(J)** in RA-T group; and 0h **(K)**, the second polar body appearance **(L)**, pronuclei appearance **(M)**, pronuclei volume is the largest and most obvious **(N)**, pronuclei disappear **(O)**, the first cleavage **(P)**, 4-cell stage **(Q)**, 8-cell stage **(R)**, morula formation **(S)** and blastocyst formation **(T)** in RA-C group.

The time-lapse monitoring (TLM) system was used to observe and record the timing of the main events of embryonic development after activation to further explore the timing of embryonic development after calcium ionophore (A23187) rescue activation. The embryos treated with calcium ionophore (A23187) rescue activation had the same developmental pattern as the normal embryos after ICSI (**Figure 1**).

**Figure 2:**
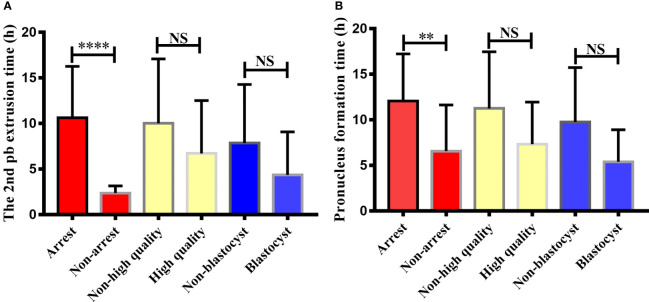
Comparison of 2nd polar body extrusion time and pronucleus formation time in different embryonic developmental groups after calcium ionophore (A23187) rescue activation. The relationship between embryonic developmental arrest, day3 embryonic status, blastocyst formation and the second polar body extrusion time and pronucleus formation time after calcium ionophore (A23187) rescue activation in unfertilized oocytes were analyzed. Non-arrest refers to embryos without developmental arrest, non-high quality refers to day3 embryo is a non-high quality embryo, non-blastocyst refers to no blastocyst formed on day 5/6. NS, no significant difference. ** indicates P < 0.01, **** indicated P < 0.0001.

The study further explored the relationship between embryo development and the timing of 2PB extrusion and pronuclei formation after calcium ionophore (A23187) rescue activation. The embryos in the RA-T group were divided into six groups: developmental arrest/non-developmental arrest, D3 high quality/non-high quality, and blastocyst formation/non-blastocyst. Then, the difference between the 2PB extrusion time and the pronuclei formation time of each group was analyzed (**Figures 2A, B**).

From the perspective of embryo development arrest, the 2PB extrusion and pronuclei formation of embryos in the non-developmental arrest group after oocyte activation were significantly earlier than those in the developmental arrest group (2.38 ± 0.21 vs 10.62 ± 0.92, *P* < 0.0001; 6.53 ± 1.53 vs 12.06 ± 1.04, *P* < 0.01). No significant difference was observed in the 2PB extrusion time and pronuclei formation time between the high-quality and poor-quality embryo groups on the D3 of embryo development (6.72 ± 1.68 vs 10.04 ± 0.95, *P* > 0.05; 7.34 ± 1.46 vs 11.26 ± 0.87, *P* > 0.05). The extrusion time of the 2PB and the pronuclei formation time in the blastocyst group were also not statistically different from those in the non-blastocyst group (4.36 ± 1.93 *vs.* 7.86 ± 1.26, *P* > 0.05; 5.41 ± 1.25 *vs.* 9.77 ± 1.19, *P* > 0.05).

### Analysis of Chromosomal Aneuploidy of Blastocysts Derived From Calcium Ionophore (A23187) Rescue Activation and Direct Activation

Biopsies were performed on seven blastocysts formed in the RA-T group, and their chromosome aneuploidy was analyzed to further clarify the chromosomal euploidy of embryos with calcium ionophore (A23187) rescue activation. Besides, the chromosomal status of the blastocysts in the RA-T group was compared with the status of 43 blastocysts in patients treated with PGT (**Table 4**). Such patients were directly activated with calcium ionophore (A23187) after ICSI because of previous ICSI fertilization failure or poor fertilization (DA-PGT group). The results showed no significant difference in the percentage of normal chromosomes between the RA-T and DA-PGT groups (42.9% vs 44.2%, *P* > 0.05). The four cases of chromosomal abnormalities in the RA-T group mainly manifested as segmental chromosome duplication, lack of an entire chromosome, uniparental disomy, and multiple abnormalities (**Supplementary Figure 1**). The abnormalities in the DA-PGT group were mainly manifested as segmental chromosome duplication or deletion, increase or deleted copy number of entire chromosomes, mosaic chromosomes.

**Table 4:** Comparison of chromosomal aneuploidy of blastocysts derived from rescue activation and direct activation of calcium ionophore (A23187).

Group	Normal	Gain	Loss	Dup	Del	Upd	Mos	Multiple abnormalities
RA-T	42.9 (3/7)	–	14.3 (1/7)	14.3 (1/7)	–	14.3 (1/7)	–	14.3 (1/7)
DA-PGT	44.2 (19/43)	2.3(1/43)	4.6 (2/43)	4.6 (2/43)	20.9 (9/43)	–	23.3(10/43)	–

Data in the table is expressed as a percentage. Gain, increased copy number of an entire chromosome; Loss, lack of an entire chromosome; Dup, segmental chromosome duplication; Del, segmental chromosome deletion; Upd, uniparental disomy; Mos, mosaic chromosomes; Multiple abnormalities, many chromosomal abnormalities are present.

## Discussion

The main reason for the failure of conventional IVF in ART is the failure of sperm penetration (18). However, ICSI avoids the process of sperm penetration into cumulus cells, sperm-oocyte penetration, and sperm-oocyte fusion. Therefore, the failed ICSI fertilization is mainly due to the failure of oocyte activation (19). The oocytes of fertilization failure are clinically discarded eventually, but these oocytes have the potential to continue to develop into embryos or blastocysts after specific treatment (20). Human embryonic stem cell lines (hESCs) can be derived from discarded human embryos. These hESCs have the potential of differentiating into multiple cell lineages and the capacity of self-renewal. It is a significant cell source for the development of regenerative medicine and changing the way of treating human diseases in the future (21, 22). Therefore, if the oocytes without fertilization after ICSI are reactivated and continue to develop, large number of available embryos will be provided for clinical usage.

Calcium ionophore (A23187) can transport Ca^2+^ from extracellular into cytoplasm, and effectively increase intracellular Ca^2+^ level (23). As a second messenger, Ca^2+^ promotes the activation of protein kinase C (PKC) and other Ca^2+^ dependent protein kinases, causing downstream protein phosphorylation and changing cell activity. In addition, calcium ionophore (A23187) can also affect cell metabolism by inhibiting mitochondrial ATPase activity and intracellular oxidative phosphorylation (24). Studies have shown that calcium ionophore (A23187) can induce the differentiation of acute myeloid leukemia into dendritic cells, affect the proliferation and cell cycle changes of hepatic stellate cells and vascular endothelial cells, and induce the sperm acrosome reaction to activate oocytes, which are now widely used in the medical field (25–27).

The widespread application of calcium ionophore (A23187) in AOA can effectively improve the outcome of the patients with sperm abnormalities, fertilization rate, implantation rate, and pregnancy rate (28–30). However, there are also some reports regarding the activation of discarded oocytes with no signs of fertilization 18–24 h post-ICSI with calcium ionophore (A23187) (31, 32). In this study, We not only systematically evaluated the rescue activation effect of calcium ionophore (A23187) on unfertilized oocytes, but also recorded and analyzed the timing of embryonic development, and performed chromosomal karyotype analysis on the blastocysts formed after rescue activation to evaluate the safety of calcium ionophore (A23187) rescue activation.

In this study, the D2 cleavage rate, D3 embryo formation rate and D3 high-quality embryo formation rate after rescue activation were significantly lower than those in the control group (58.5% *vs* 98.0%, 50.0% *vs* 89.1%, 33.3% *vs* 60.1%; *P* < 0.05). Some researchers used calcium ionophore (A23187) to activate unfertilized oocytes 24 h post-ICSI, and obtained 72.5% and 62.7% of fertilization rate and cleavage rate, the high-quality embryo rate was 11.8% (33). Their results were consistent with our study, indicating that calcium ionophore (A23187) could partially restore the continuous development of unfertilized oocytes after ICSI.

Some researchers also used calcium ionophore (A23187) combined with ionomycin to activate unfertilized oocytes 18 h post-ICSI *in vitro (* 34). They found that 84.9% of oocytes were successfully activated, the normal fertilization rate and cleavage rate was 30.1% and 64.0%, respectively. However, most embryo after cleavage can only develop to the 4-cell stage (34). Economou et al. (35) evaluated the activation effect of applying calcium ionophore (A23187) alone and calcium ionophore (A23187) combined with GM-CSF on unfertilized oocytes 18h post-ICSI, then performed karyotype analysis of the formed blastocysts. Compared with our study, their study showed a lower fertilization rate and lower D3 quality embryo formation rate, but a higher normal fertilization rate (76.2% *vs* 43%, 50.0% *vs* 19%, 40.6% *vs* 79%; *P*<0.05). In their study, calcium ionophore (A23187) which was used alone did not obtain blastocysts with normal chromosomal karyotypes, and the blastocyst formation rate in our study was higher than their study (66.7% *vs* 13.0%; *P* < 0.05), which may be caused by differences in embryo culture conditions, treatment methods, and individual differences in the included patients. In addition, they also found that the combined use of calcium ionophore (A23187) and GM-CSF activation resulted in higher fertilization rates, high-quality embryo formation rates, and blastocyst formation rates than using calcium ionophore (A23187) alone. Perhaps in future studies the combined use of calcium ionophore (A23187) and GM-CSF activation treatment will be more beneficial to the embryonic development after activation (35).

In our study, the total fertilization rate in the RA-T group was slightly higher than that in the RA-C group, the normal fertilization rate was lower than that in the control group, and the abnormal fertilization rate was higher than that in the control group, indicating that although calcium ionophore (A23187) could partially activate the developmental potential of oocytes, it might also increase the occurrence of abnormal fertilization or due to the abnormal of the oocyte itself.

The results of the present study showed no significant difference in the blastocyst formation rate and high-quality blastocyst formation rate between the RA-T and RA-C groups. The blastocyst formation rate and the high-quality blastocyst formation rate in the RA-T group were even slightly higher than those in the RA-C group. This might be due to the high-quality embryos was usually clinically used for transferring or freezing; the embryos for blastocyst culture were not the best-quality embryos and sometimes even low-quality embryos not suitable for transfer or freezing.

Studies have reported that the use of calcium ionophore (A23187) rescue activation of oocytes 24h post-ICSI, transplantation of the embryos formed, and successfully delivered babies with normal chromosomal karyotypes (36). Nevertheless, the transfer of such embryos still needs to be carefully selected. From the current research results, one patient who underwent embryo freeze-thaw transfer received a live birth last year in RA group, and calcium ionophore (A23187) can obtain blastocysts with normal karyotype for rescue activation of unfertilized oocytes after ICSI injection in RA-T group, but whether it may affect the epigenetic changes that affect embryonic development is still unknown, long-term follow-up studies are needed for the offspring who have been born. In addition, it is necessary to further increase the research of animal experiments, especially the detailed evaluation of the weight, intelligence, organ development and reproductive ability of the offspring.

By using TLM system, the timing of different development types of embryos in the RA-T group were compared. The results showed that the 2PB extrusion time and the PN formation time of oocytes from normal embryos (i.e., without developmental arrest) after activation with calcium ionophore (A23187) were significantly earlier than those in the arrest group. Payne et al. (37) reported that 2PB extrusion occurred 1–8 h post-ICSI, with an average of 2.65 h, while the formation time of PN was 2–12 h post-ICSI, with an average of 4.98 h (37). In this study, the average extrusion time of 2PB was 2.38 h in the RA-T group, which was consistent with the results reported by Payne et al. (37). The average time of PN formation in the RA-T group was 6.53 h, slightly later than the 4.98 h reported by Payne et al. (37), which might be related to the slow formation of PN caused by the delayed oocyte activation. Besides, they also found that the extrusion time of 2PB from high-quality embryos was shorter than that of non-high-quality embryos (2.45 h *vs* 3.23 h, *P* = 0.03). However, in this study, no significant differences in 2PB extrusion time and PN formation time between the high-quality and non-high-quality embryo groups, and the blastocyst formation and non-blastocyst formation groups. In addition, the 2PB extrusion time and PN formation time in the high-quality embryo and blastocyst formation groups in RA-T group were shorter than those in the non-quality embryo and no blastocyst formation groups.

Four couples with a history of incomplete ICSI cycles owing to 2PN arrest and undergo a subsequent ICSI cycle combined with AOA using calcium ionophore (A23187) were collected by Darwish et al. (38), and 2 healthy babies were delivered (38). In their study, the fertilization rate and cleavage rate after direct activation of calcium ionophore (A23187) were 67.6% and 44%, respectively, which were significantly higher than those in the previous cycle. The result is comparable to the fertilization rate and cleavage rate of DA group in our study (58.3% and 37.9%), and a total of 18 healthy babies were delivered in DA group. These results indicating that direct activation of calcium ionophore (A23187) can improve the outcome of patients with complete fertilization failure. A study analyzed the effect of calcium ionophore (A23187) combined with ionomycin on the activation of non-fertilized oocytes at different time points (20–68 h) post-ICSI. The results showed that the developmental potential of oocytes after activation gradually decreased with delayed activation, and the optimal time for rescue activation was 20 h post-ICSI (39). In this study, the early cleavage rate, cleavage rate, high-quality embryo rate, blastocyst formation rate and high-quality blastocyst rate in the DA group were significantly higher than those in the RA group (37.9% *vs* 4.2%, 96.9% vs 89.6%, 56.9% *vs* 23.3%, 44.0% *vs* 10.5%, 14.3% *vs* 0.00%, *P* < 0.05). These results indicated that the developmental potential of oocytes directly activated after ICSI was better than that of oocytes activated on the next day. Therefore, calcium ionophore (A23187) should be used as early as possible to improve the quality of the embryos.

There was also a study found that the fertilization rate, embryo quality, implantation rate, and live birth rate of rescue ICSI on the next day after fertilization failure in conventional overnight IVF cycles were significantly lower than those for oocytes with early rescue activation and conventional IVF with successful fertilization (40). This was consistent with the results that the fertilization and embryo development in the RA group were worse than those in the DA group, which might be caused by oocyte aging. Compared with the rescue ICSI on the next day after fertilization failure in conventional overnight IVF cycles, calcium ionophore (A23187) rescue activation could significantly increase the percentage of normal fertilization (62.3% *vs* 30.4%, P < 0.05) (41). Therefore, for the oocytes that failed in conventional overnight IVF, the rescue ICSI combined with calcium ionophore (A23187) activation might improve the inefficiency of rescue ICSI caused by oocyte aging to some extent, thus improving the fertilization rate and embryo development ability.

Through further analysis of the clinical data, a total of 77 unfertilized oocytes in the RA group were collected for calcium ionophore (A23187) rescue activation. As a result, 10 high-quality embryos and 32 non-high-quality embryos were obtained. Among them, 4 high-quality embryos were frozen, and the remaining 6 high-quality embryos and 32 non-high-quality embryos continued to be cultured. Four non-high-quality blastocysts were obtained. Because the development of embryo derived from calcium ionophore (A23187) rescue activation is not synchronized with the development of endometrial, the embryos derived from the rescue activation are not freshly transferred in this cycle, and the formed blastocysts are frozen. After further analysis of the results of freeze-thawing transfer in the RA group, it was found that 4 non-high-quality blastocysts were thawed and transferred without pregnancy. However, one patient who underwent embryo freeze-thaw transfer received a live birth last year and is currently in normal health. Nevertheless, the embryonic development quality of the rescue activation group is not as good as that of the direct activation group, which may be related to some problems in these embryos.

The present study found that despite no significant difference in the fertilization rate between the RA-T and RA groups (76.2% *vs* 67.5%, *P* > 0.05), the cleavage rate and embryonic development ability in the RA group were lower than those in the RA-T group. This might be related to the small sample size of the RA group. On the contrary, it might also be related to the complete fertilization failure after ICSI caused by abnormal fertilization in the RA group. In addition, the levels of LH and AMH in the RA group were significantly different from those in the other groups, suggesting that the ovarian function and oocyte quality in the RA group was inferior to those in the RA-T group with relatively normal fertilization ability, which might be the factors affecting the cleavage and embryo development ability in the RA group (42–44).

The application of calcium ionophore (A23187) can increase the fertilization rate and improve the outcome of embryo development. However, the early stage of embryo development is easily affected by environmental factors, resulting in development arrest or chromosomal abnormalities. Therefore, the safety of oocyte activation by calcium ionophore (A23187) might a concern for researchers. Several studies reported that oocytes treated with a low concentration of calcium ionophore (A23187) resulted in healthy babies, and no abnormal development of babies at birth was observed (45, 46). Kyono et al. (40) conducted a 4-year follow-up on babies treated with calcium ionophore (A23187) and found no developmental abnormalities (47). In this study, no statistically significant difference was reported in the euploidy of blastocyst chromosomes between the RA-T and DA-PGT groups (42.9% *vs* 44.2%, *P* > 0.05). The chromosomal abnormalities of blastocysts in the RA-T group were Loss (14.3%), Dup (14.3%), and Upd (14.3%), while the chromosomal abnormalities of blastocysts in the DA-PGT group were mainly Mos (23.3%) and Del (20.9%). Since the SNP detection method was adopted for the chromosome analysis in the RA-T group, the uniparental disomy could be detected. However, the next-generation sequencing method was adopted in the DA-PGT group, and hence triploid and uniparental disomy could not be detected. However, there was also a certain percentage of blastocysts with normal karyotype in the RA-T group. Previously, SNP was used to detect the chromosome euploidy of blastocysts formed from clinically discarded 2PN-derived embryos, and a certain percentage of blastocysts with normal chromosomes were also obtained. No significant difference in the normal chromosome percentage of blastocysts in the RA-T group was observed in this study (60.7% *vs* 42.9%, *P* < 0.05). This result showed that the short-term treatment of oocytes with calcium ionophore (A23187) did not significantly affect the chromosome euploidy.

## Conclusions

In conclusions, the present study showed that the unfertilized oocytes 18-24 h post-ICSI could continue to develop after the activation of calcium ionophore (A23187), and a certain proportion of high-quality blastocysts with normal karyotype could be obtained. For patients whose oocytes are completely unfertilized or poorly fertilized, calcium ionophore (A23187) was supposed to activate as soon as possible after ICSI to increase the fertilization rate and improve embryonic development potential. In this way, at least some patients can get usable embryos during this cycle, reducing the patient’s financial burden and waiting time. Chromosome analysis of the blastocyst obtained after calcium ionophore (A23187) rescue activation can obtain blastocysts with normal karyotypes, which provides some evidence for the widely use of calcium ionophore (A23187) rescue activation in reproductive clinics in the future.

## Data Availability Statement

The original contributions presented in the study are included in the article/**Supplementary Material**. Further inquiries can be directed to the corresponding authors.

## Ethics Statement

The studies involving human participants were reviewed and approved by Life Science and Ethics Review Committee of the First Affiliated Hospital of Zhengzhou University. The patients/participants provided their written informed consent to participate in this study.

## Author Contributions

ZX: performing the experiment, data interpretation, data analysis, and drafting the article. GY: conception and design the study, performing the experiment, acquisition of data, and data analysis. WN and HF: performing the experiment, data interpretation, and data analysis. XM, SS, HJ, and WS: collecting samples. YS: conception and design the study. All authors participated in the revision of the manuscript. All authors contributed to the article and approved the submitted version.

## Funding

This work was supported by grants from the National Natural Science Foundation of China (U1904138 to GY, 81820108016 to YS).

## Conflict of Interest

The authors declare that the research was conducted in the absence of any commercial or financial relationships that could be construed as a potential conflict of interest.
