# Supplementary material for: Calcium Ionophore (A23187) Rescues the Activation of Unfertilized Oocytes After Intracytoplasmic Sperm Injection and Chromosome Analysis of Blastocyst After Activation
Source: Front Endocrinol (Lausanne). 2021 Jul 15;12:692082. doi: 10.3389/fendo.2021.692082 (PMC8320372; doi:10.3389/fendo.2021.692082)
Supplement: Supplementary file 1 [file DataSheet_1.docx]

**B1: 45, XX, -13, multiple uniparental disomy (UPD)**


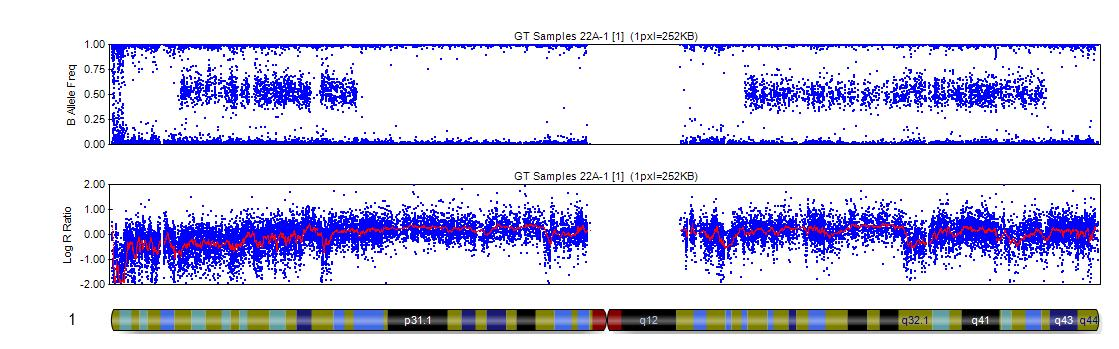


Chromosome 1: UPD


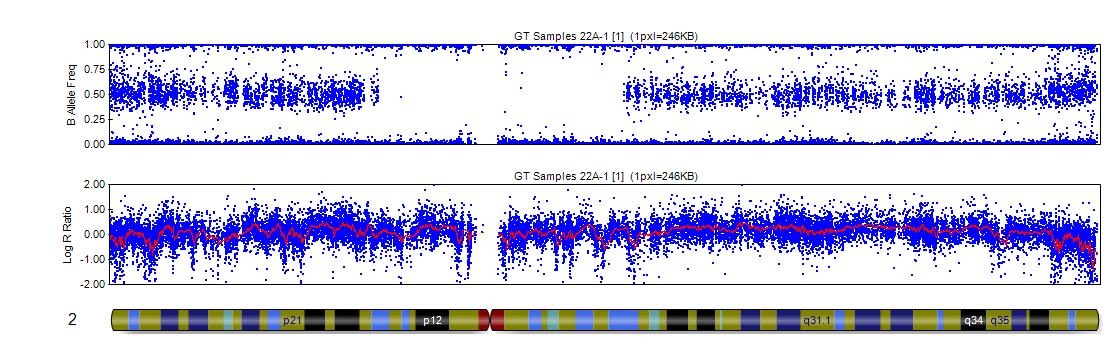


Chromosome 2: UPD


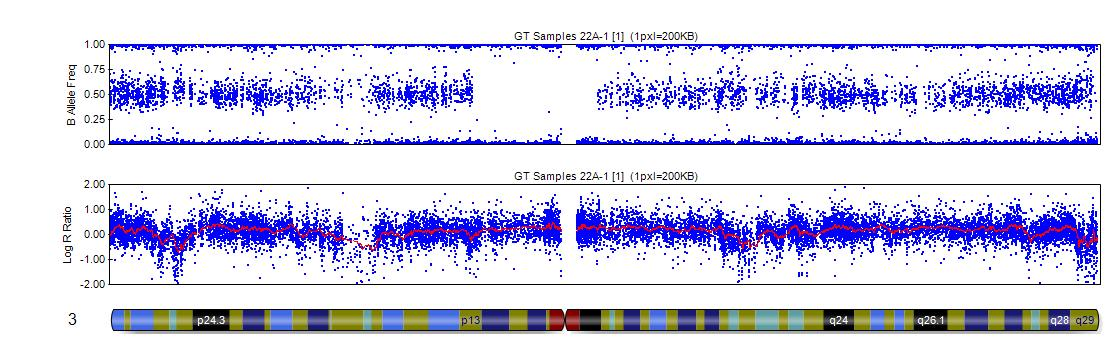


Chromosome 3: UPD


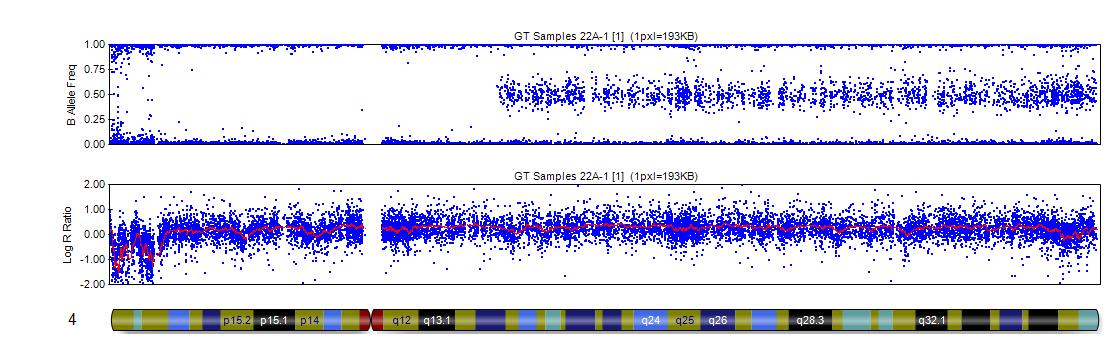


Chromosome 4: UPD


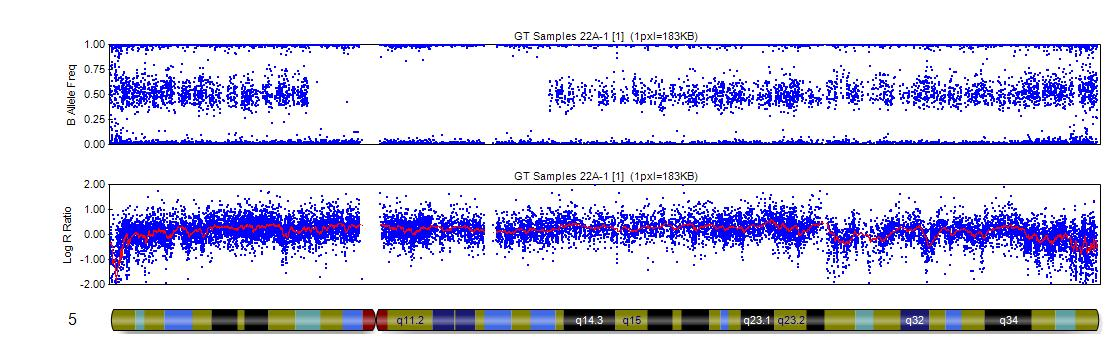


Chromosome 5: UPD


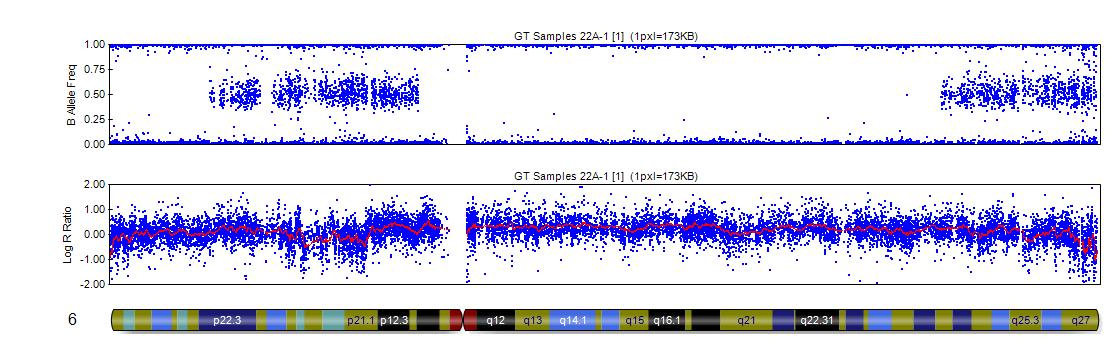


Chromosome 6: UPD


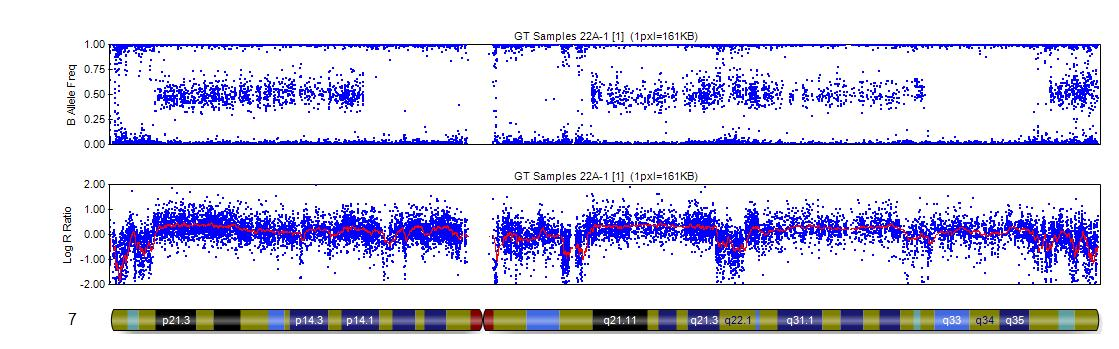


Chromosome 7: UPD


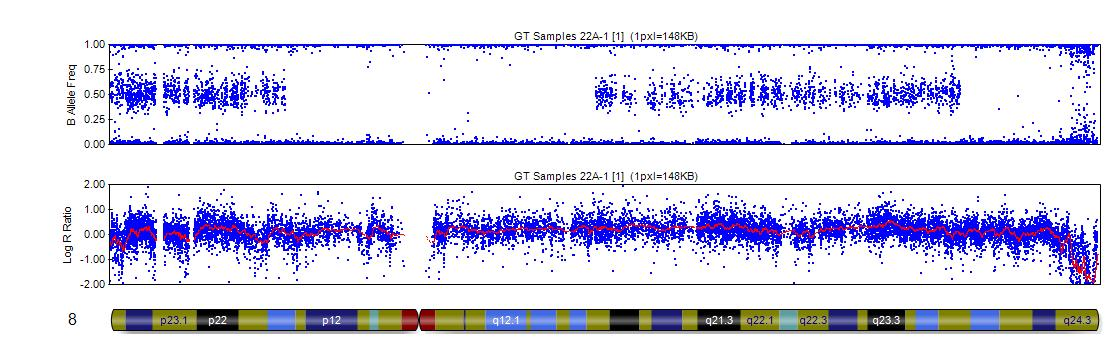


Chromosome 8: UPD


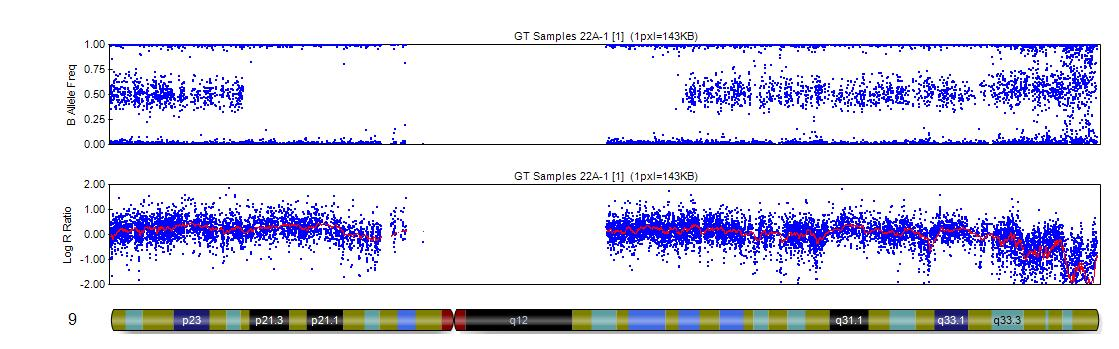


Chromosome 9: UPD


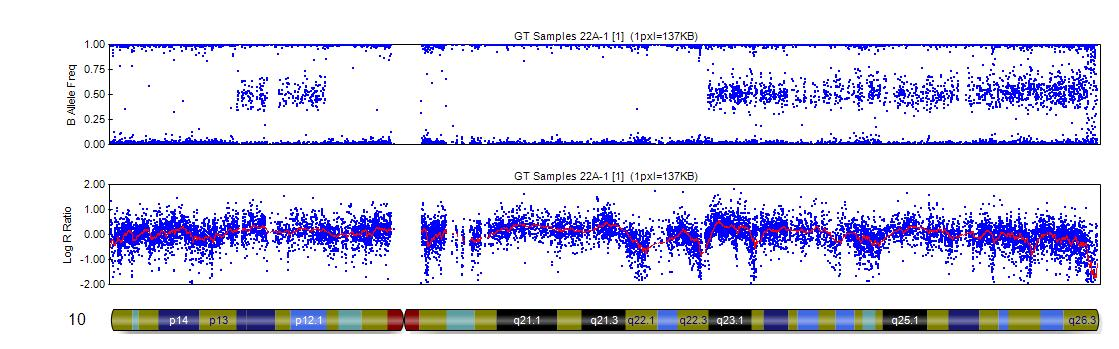


Chromosome 10: UPD


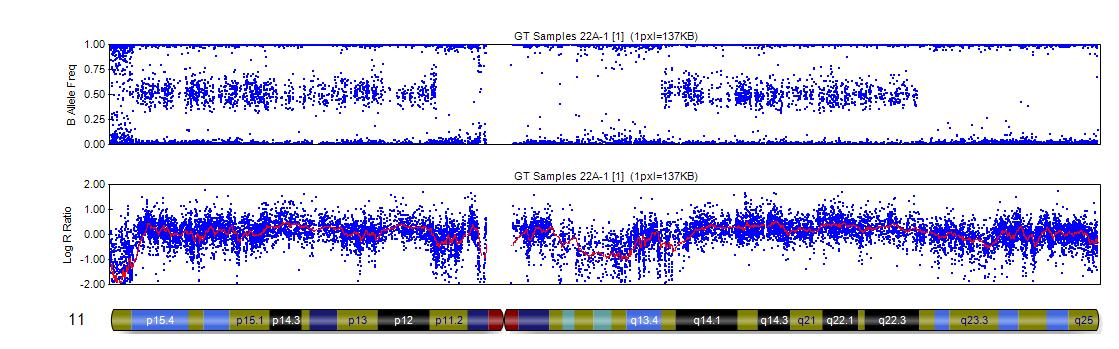


Chromosome 11: UPD


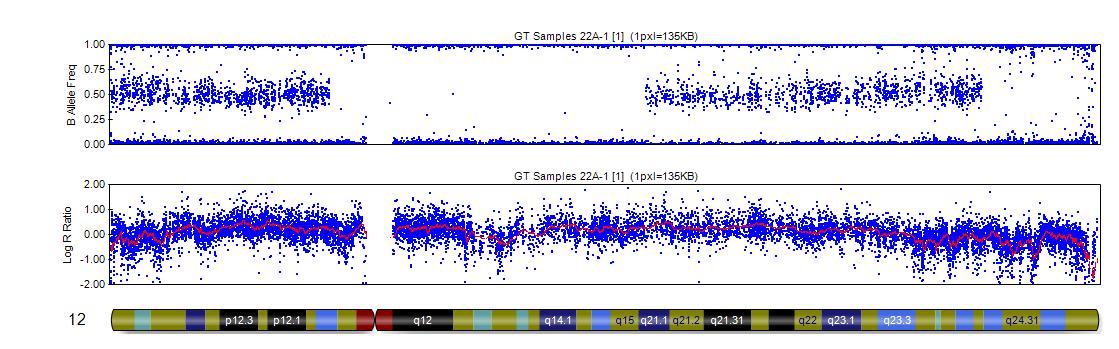


Chromosome 12: UPD


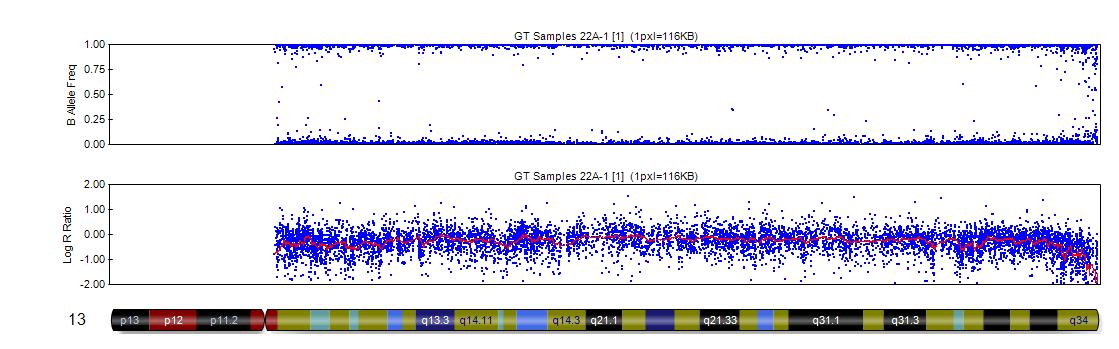


Chromosome 13: chromosome monomer


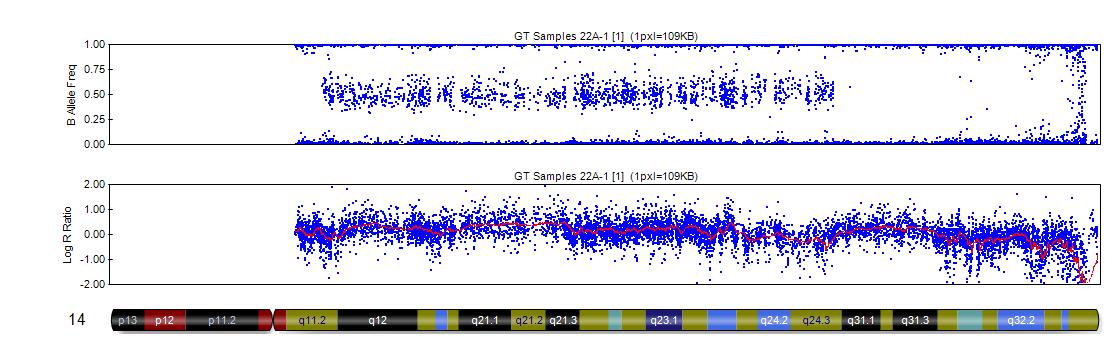


Chromosome 14: UPD


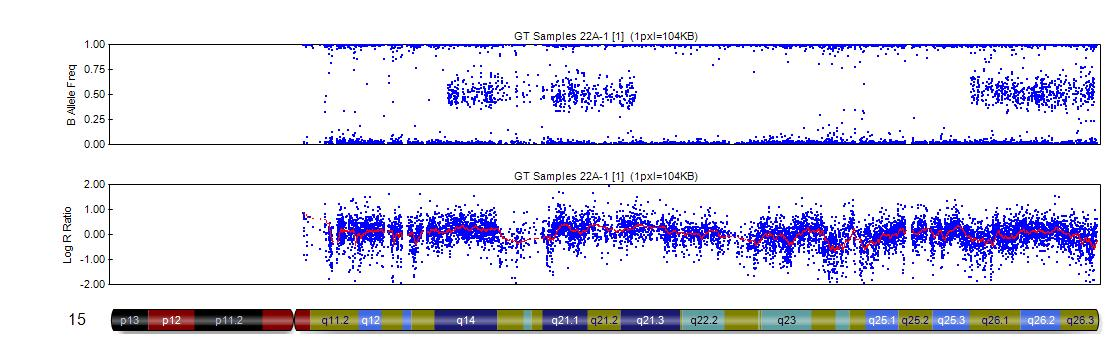


Chromosome 15: UPD


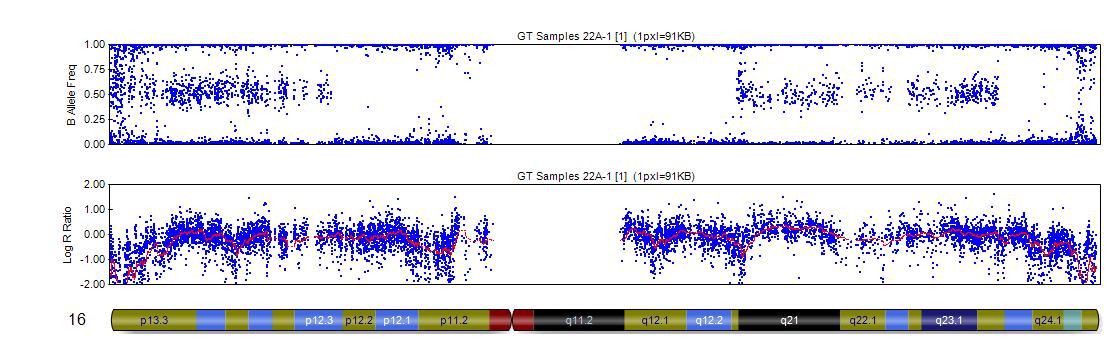


Chromosome 16: UPD


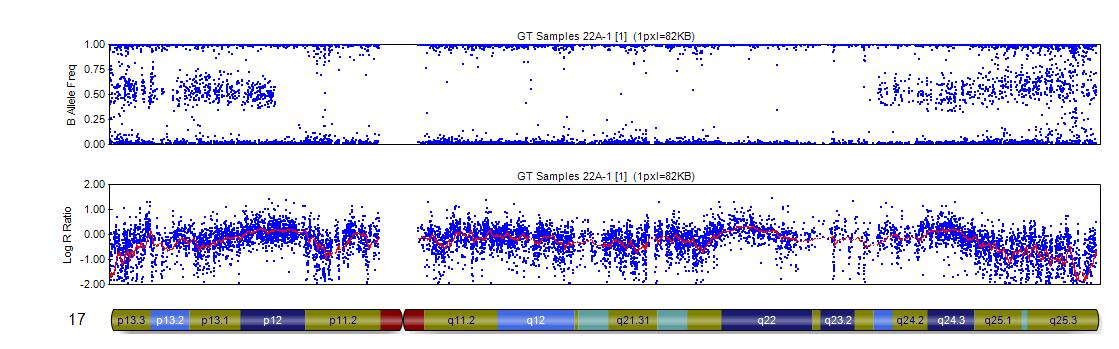


Chromosome 17: UPD


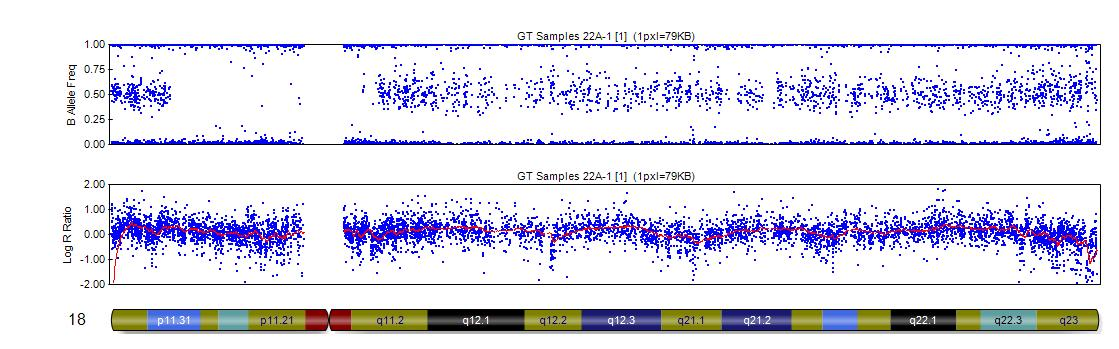


Chromosome 18: UPD


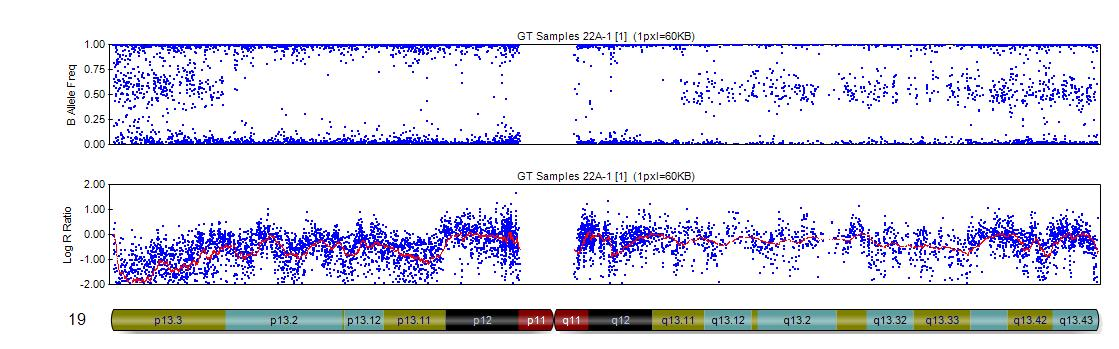


Chromosome 19: UPD


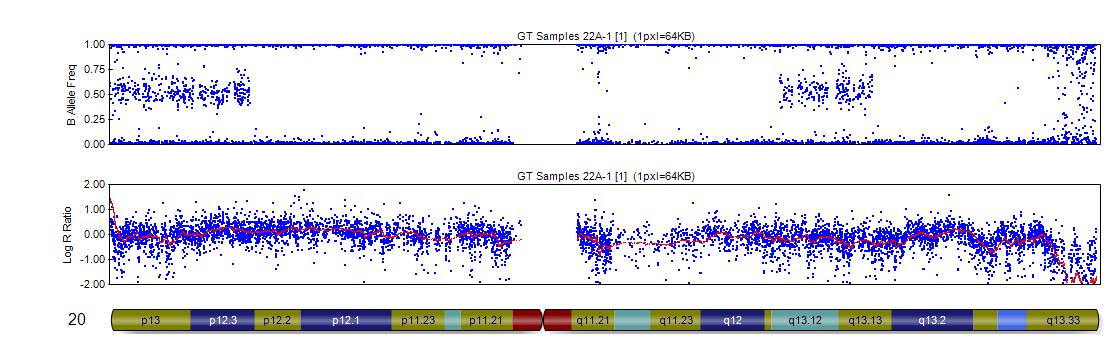


Chromosome 20: UPD


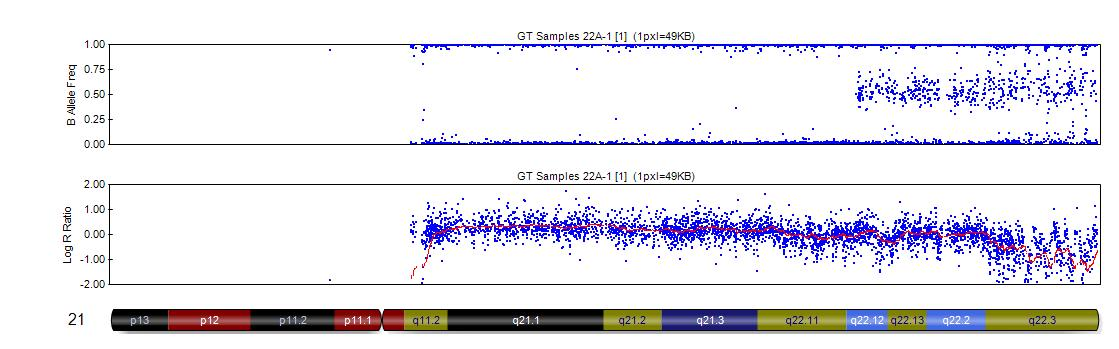


Chromosome 21: UPD


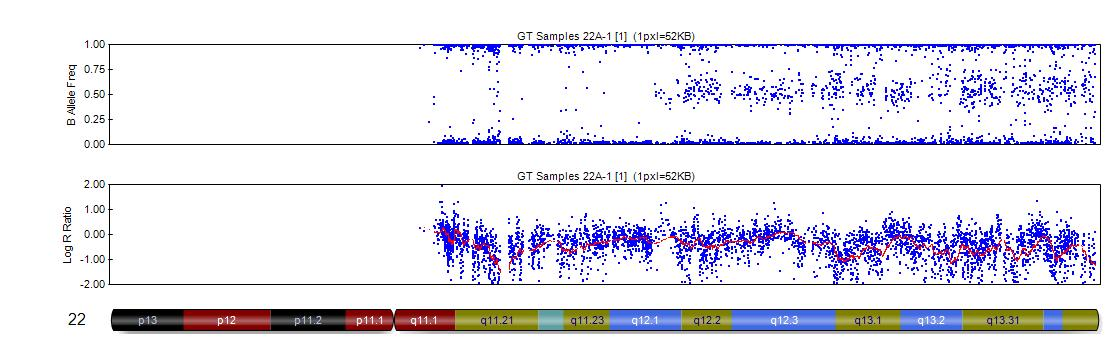


Chromosome 22: UPD


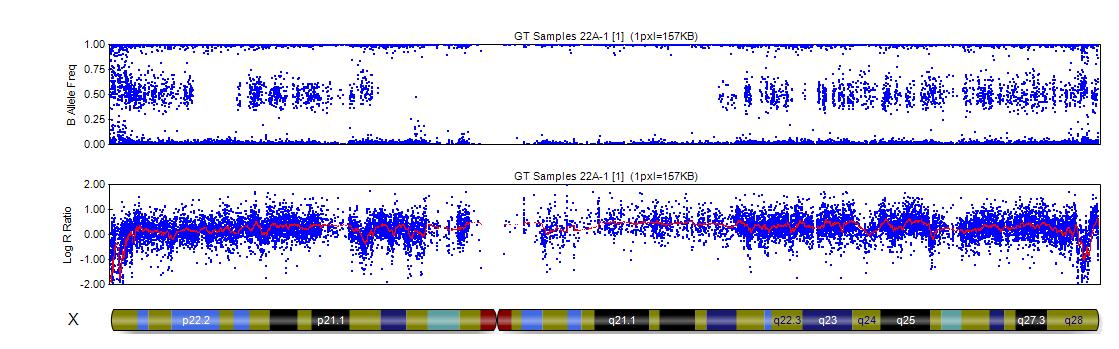


Chromosome X: UPD

**B2: dup (1) (p32.244), +2, +3, +4, +5, +6, +7, +8, +9, +10, +11, +12, +15, +16, +17, +18, +19, +20, upd (21) (q11.2-q21.3), +22, XXY**


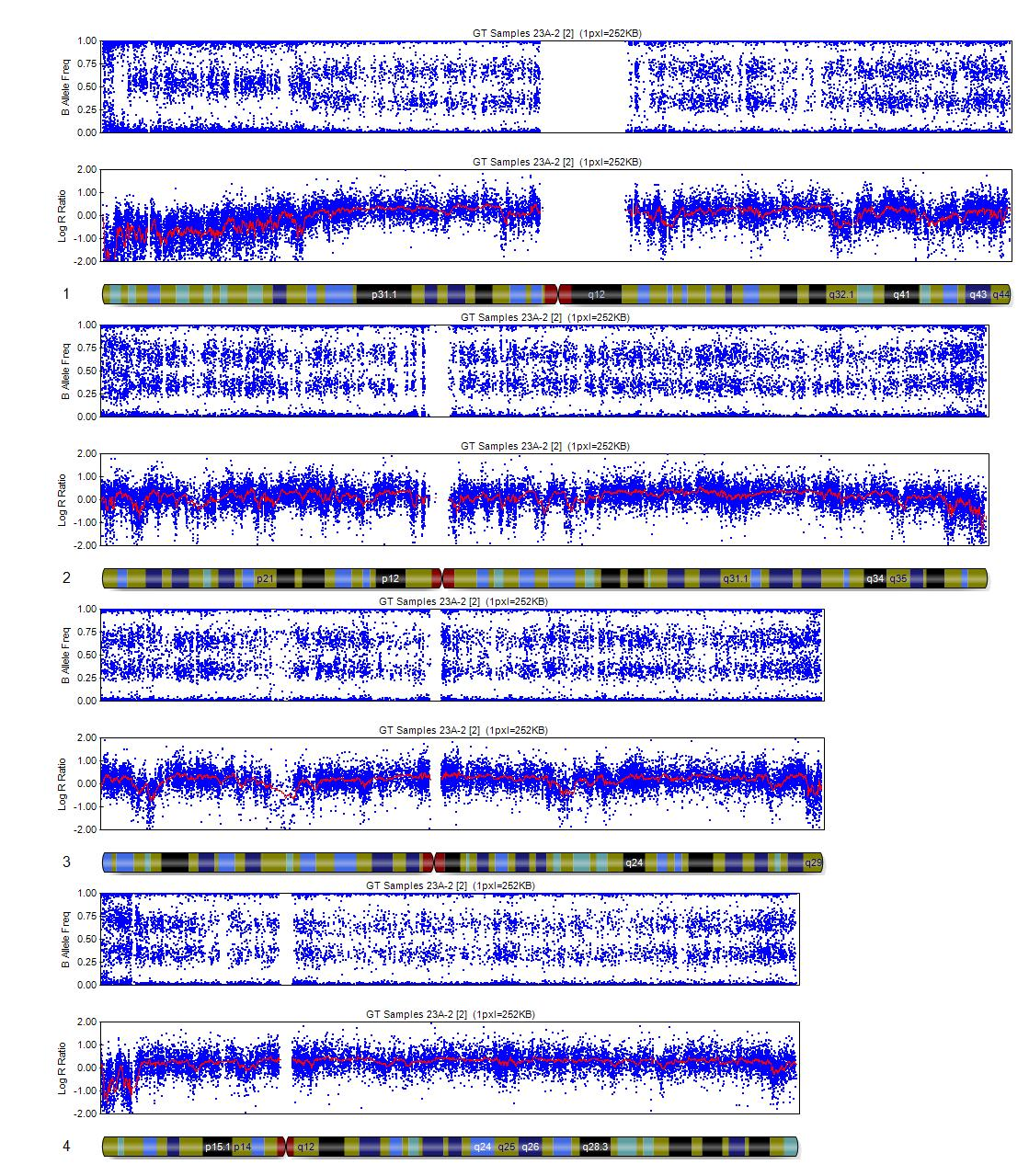

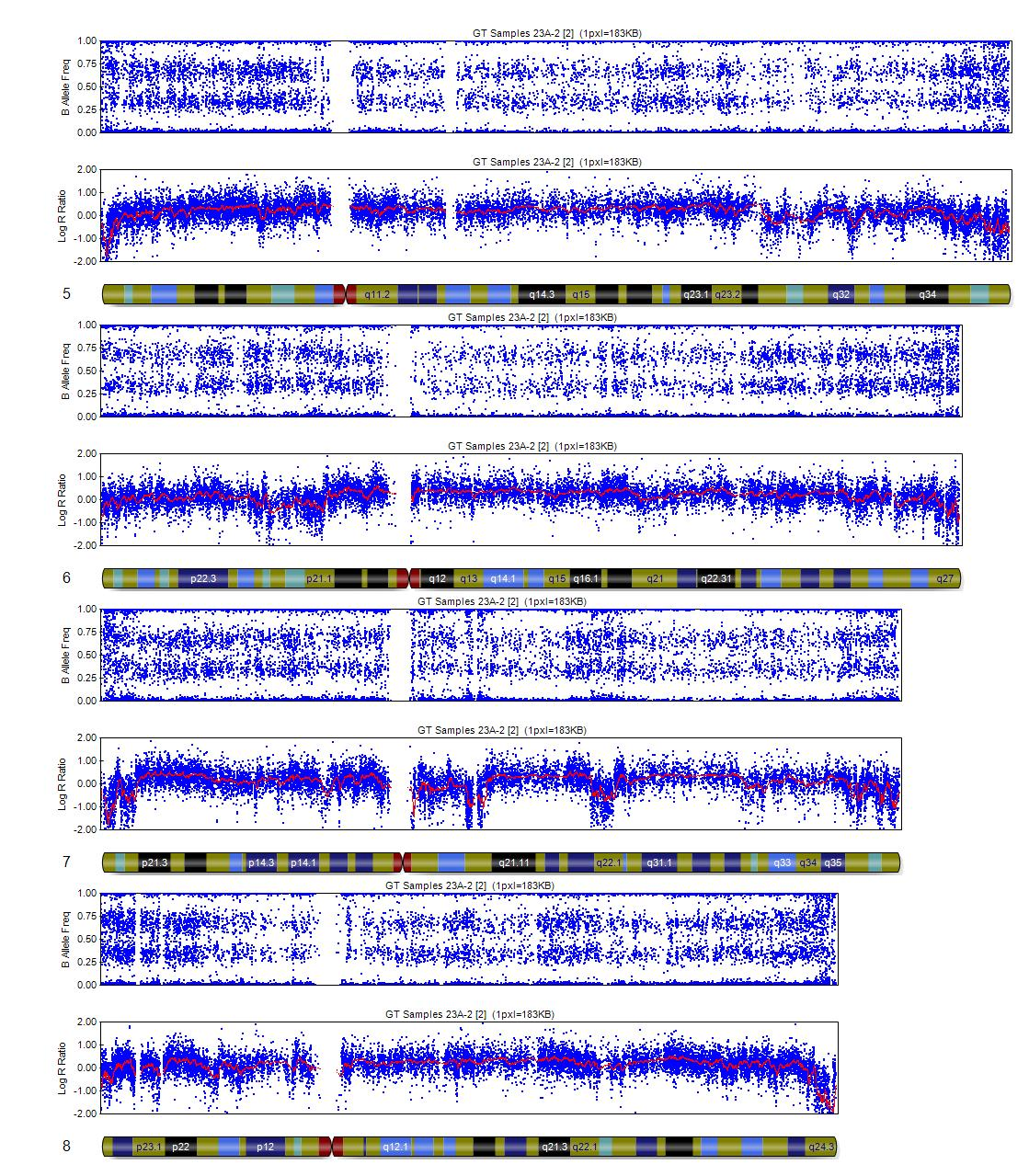

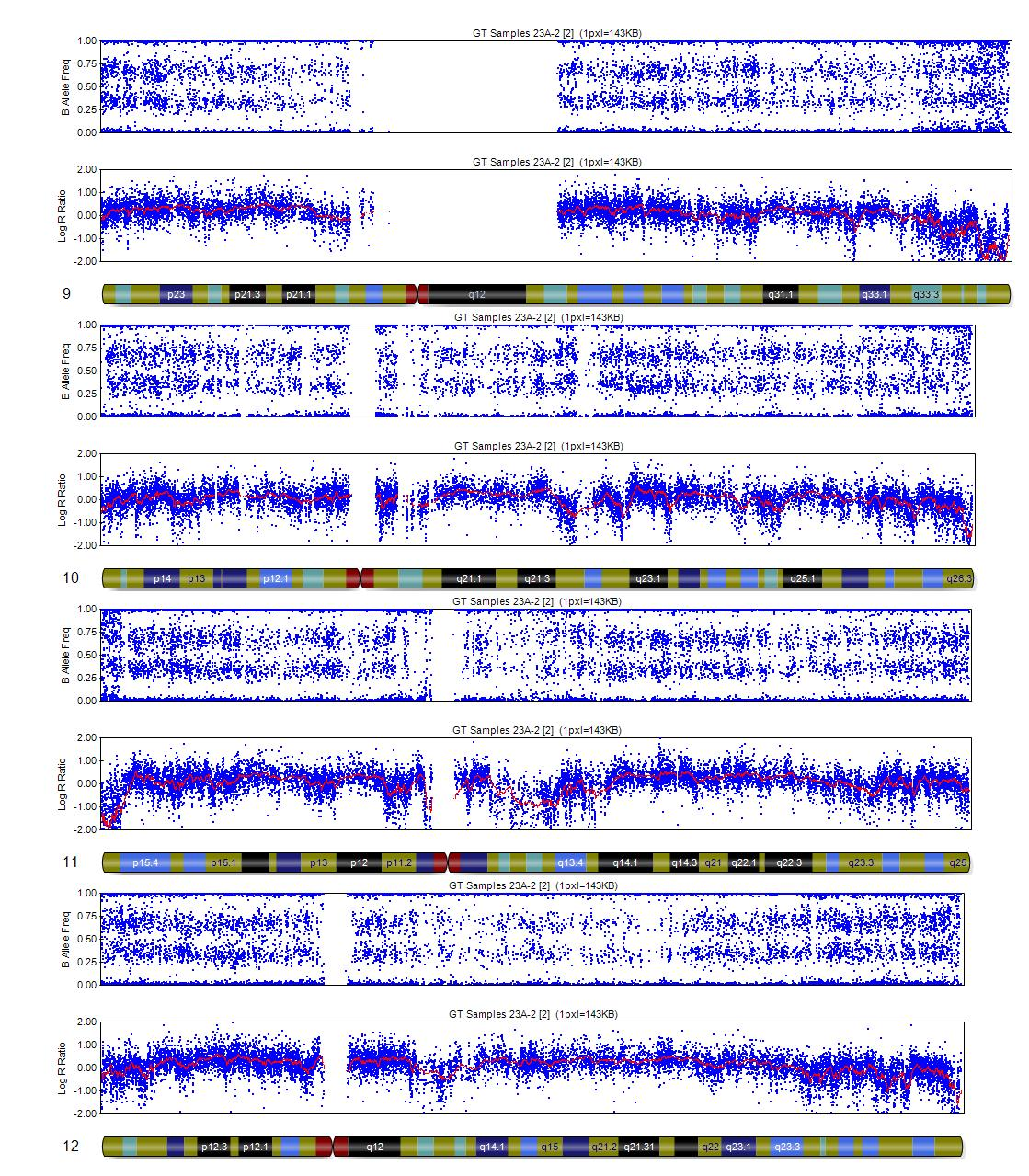

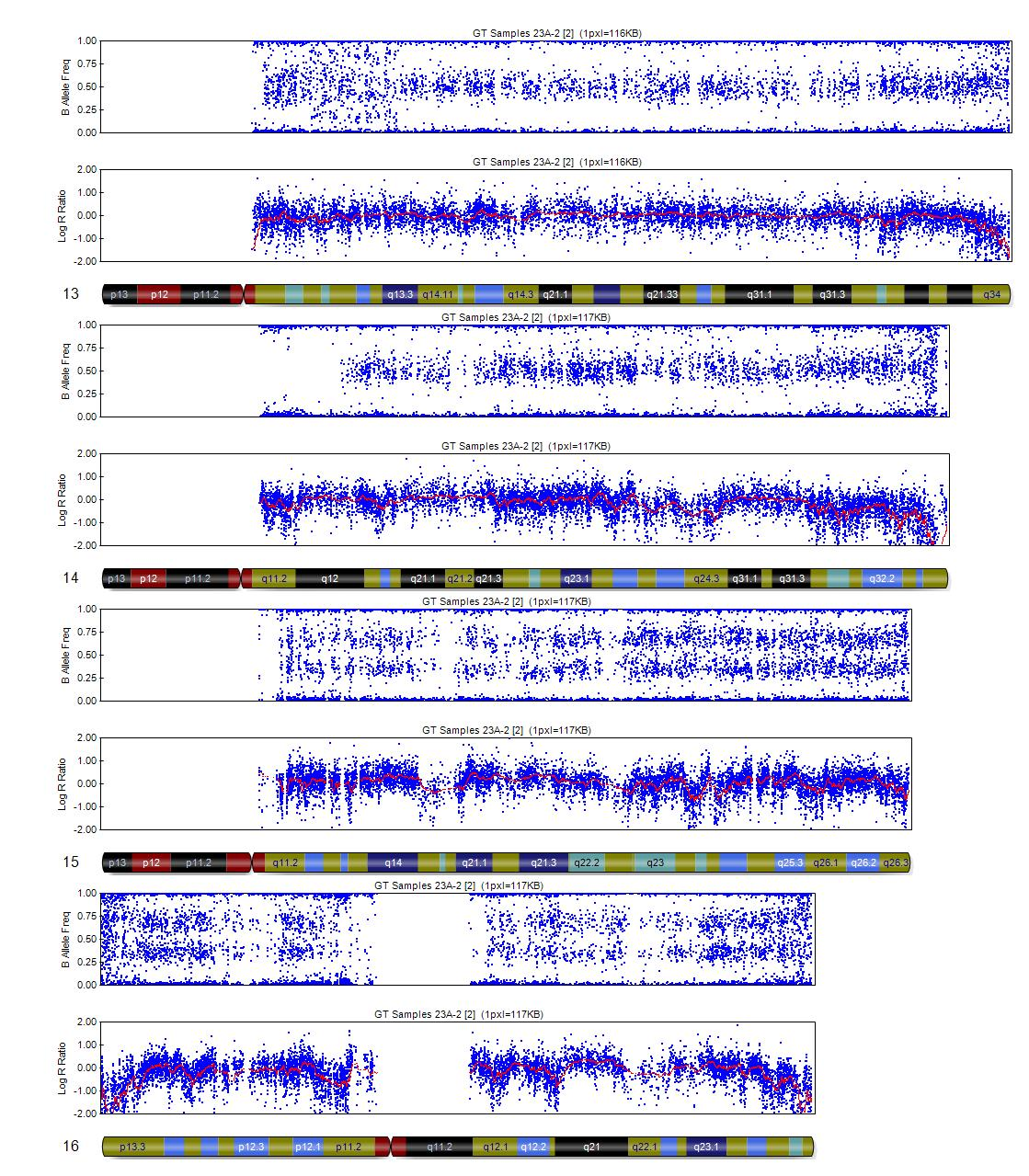

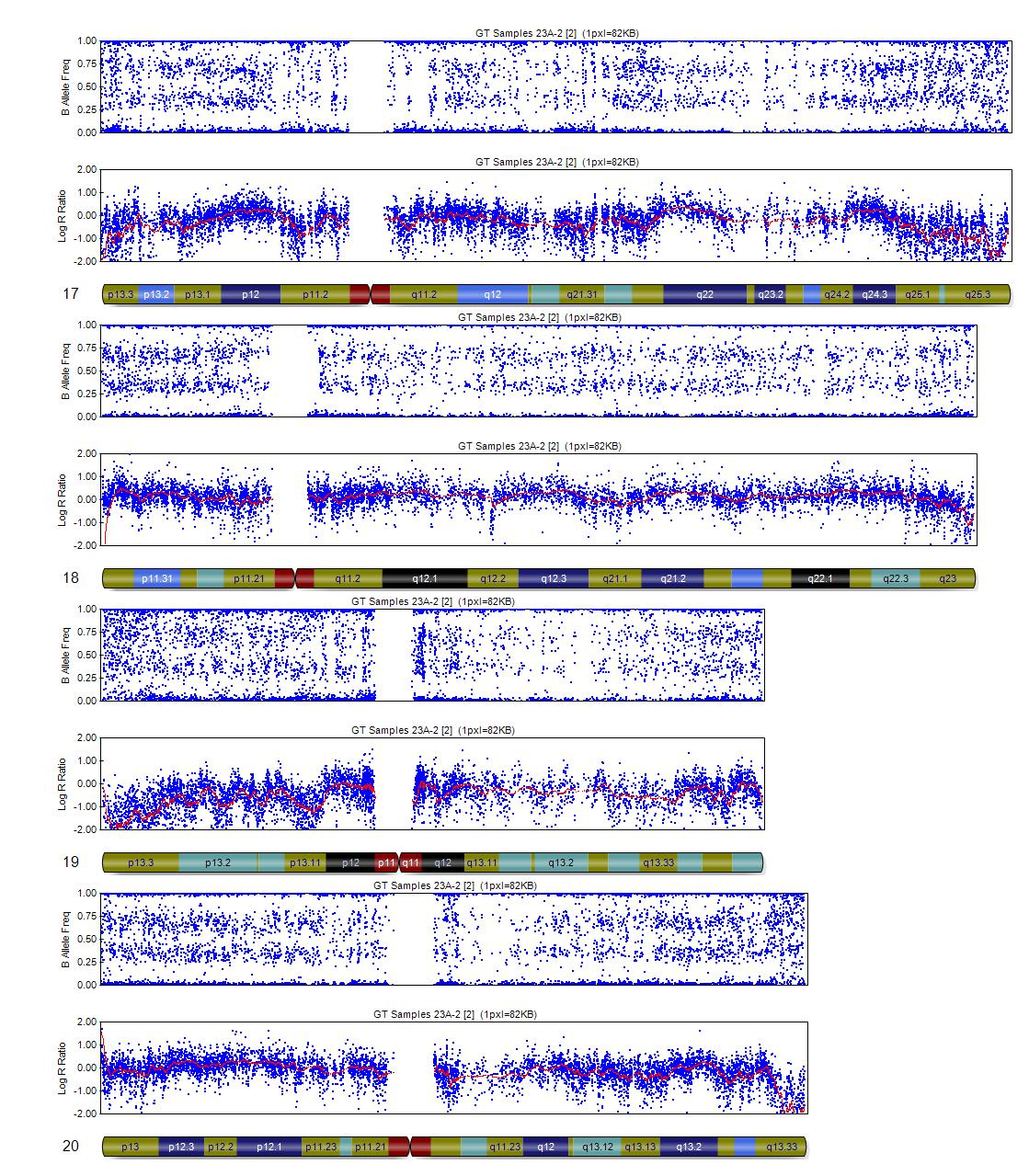


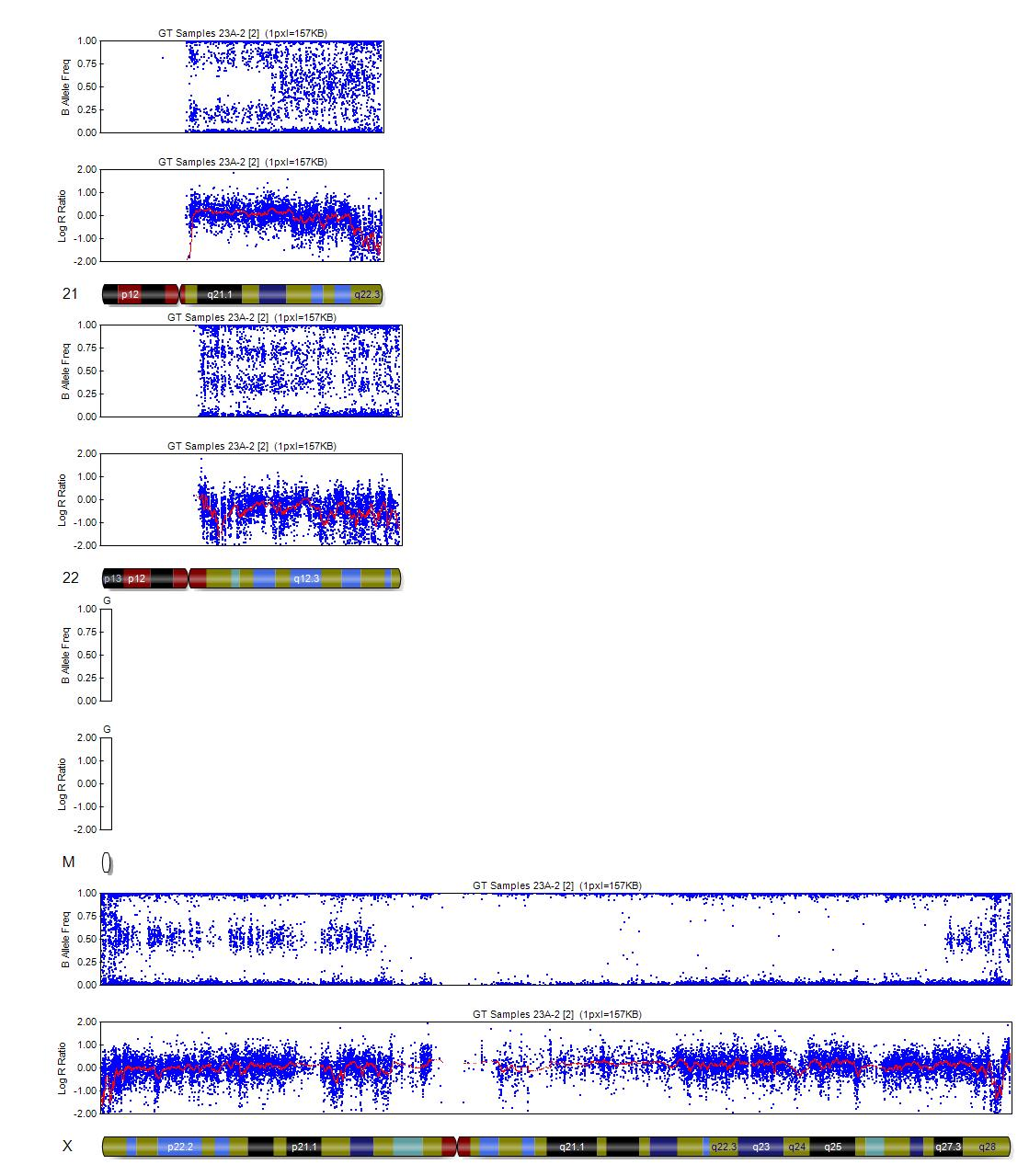

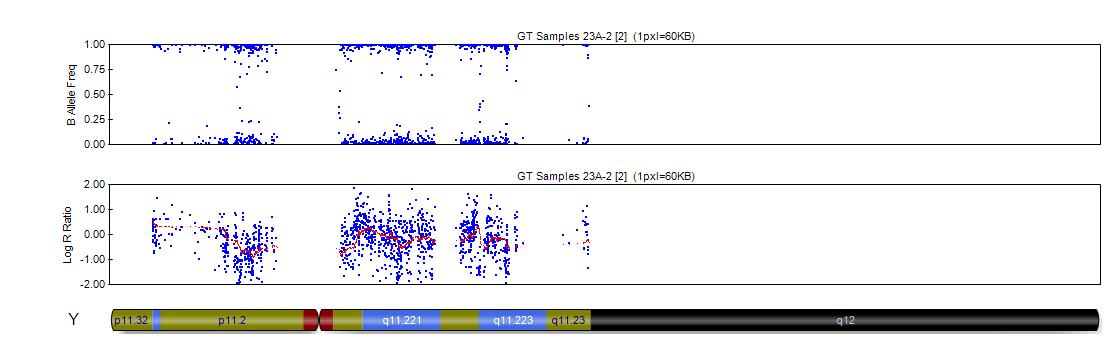


**B3: 46, XY**


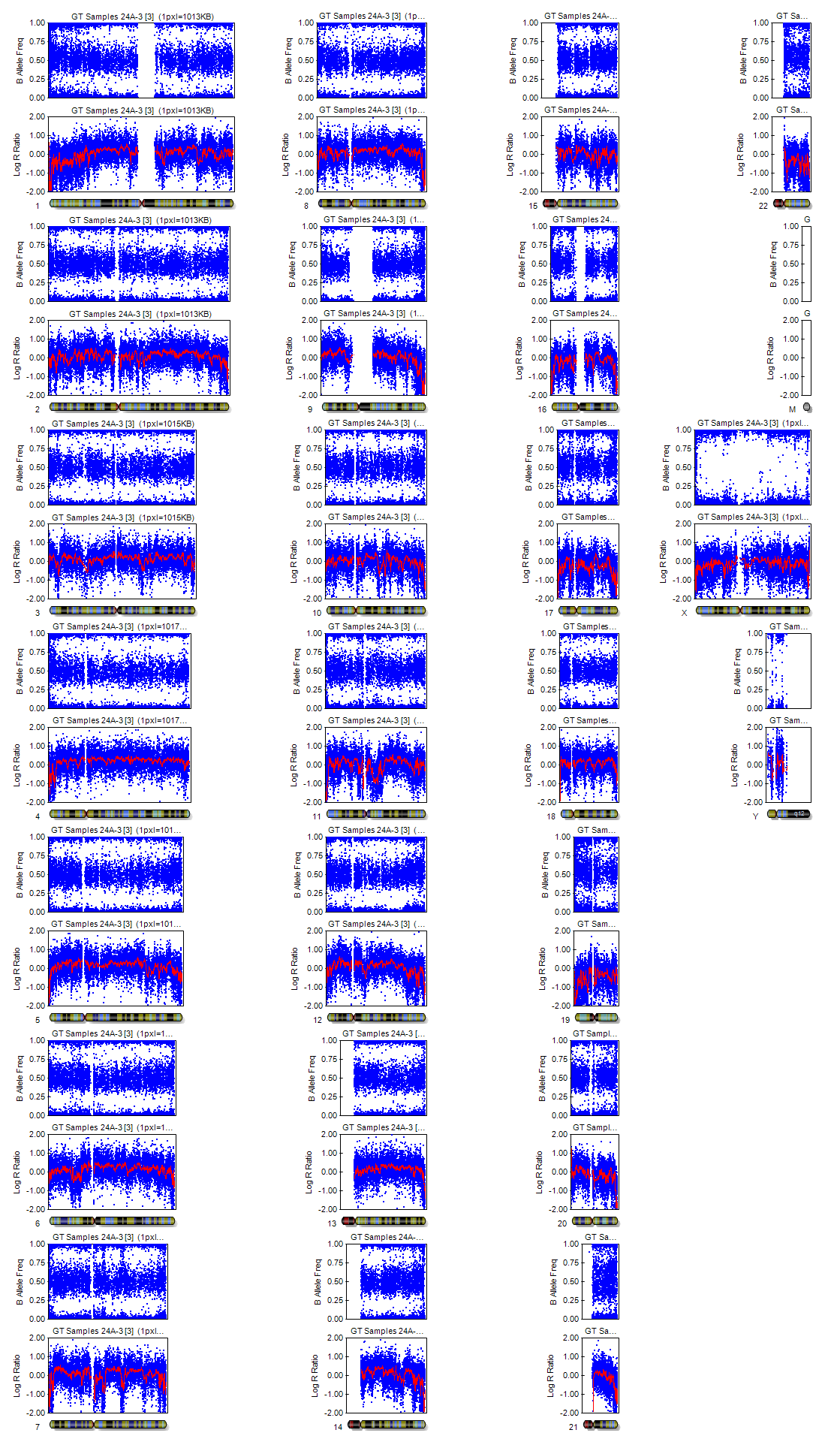


**B4: 45, XX, -14, dup (10) (pter-q22.2）, del (10) (q22.3-q26.3)**


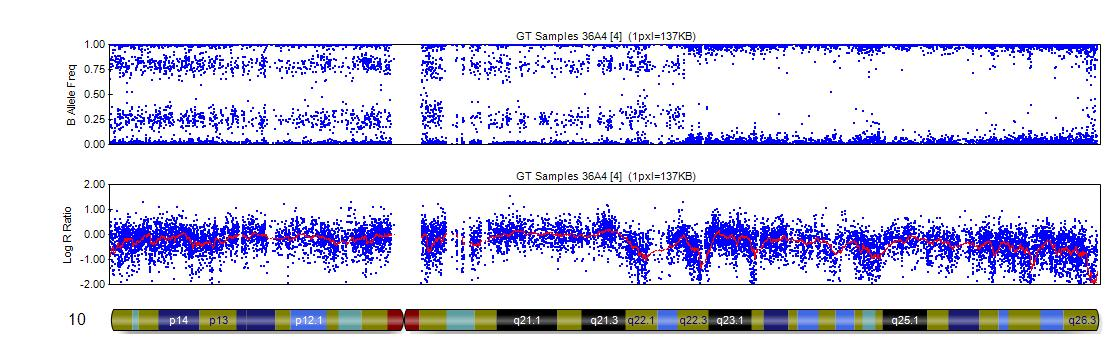


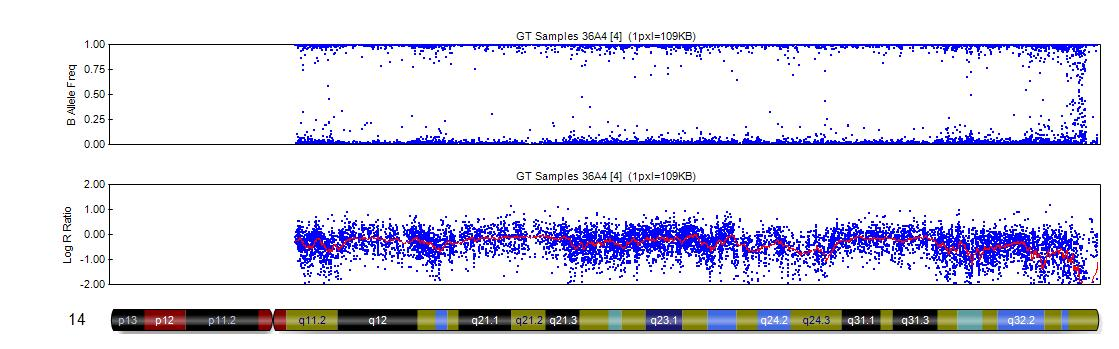


**B5: 46, XX**


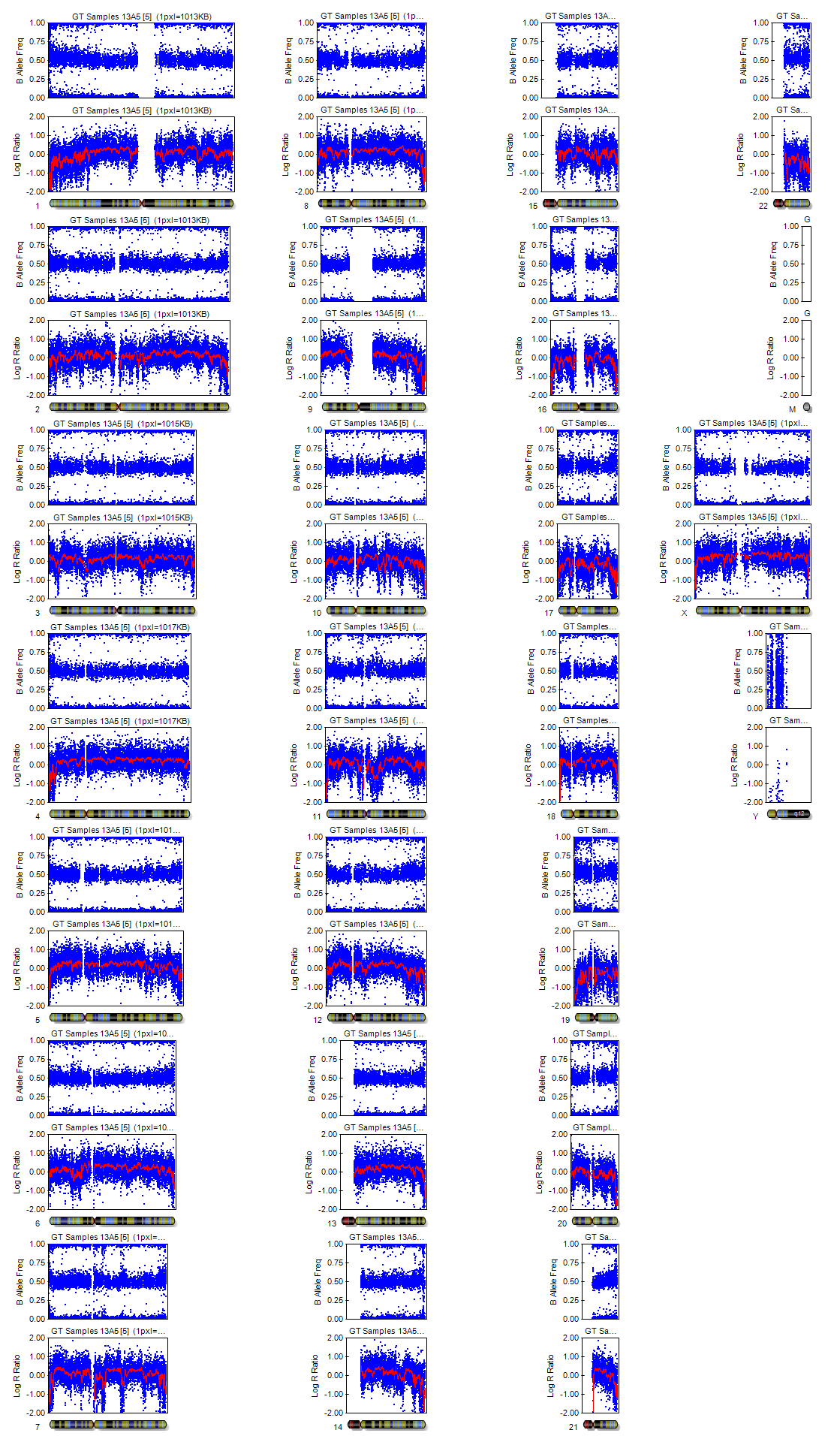


**B6: 46, XX, dup(2)(pter-p23.1)**


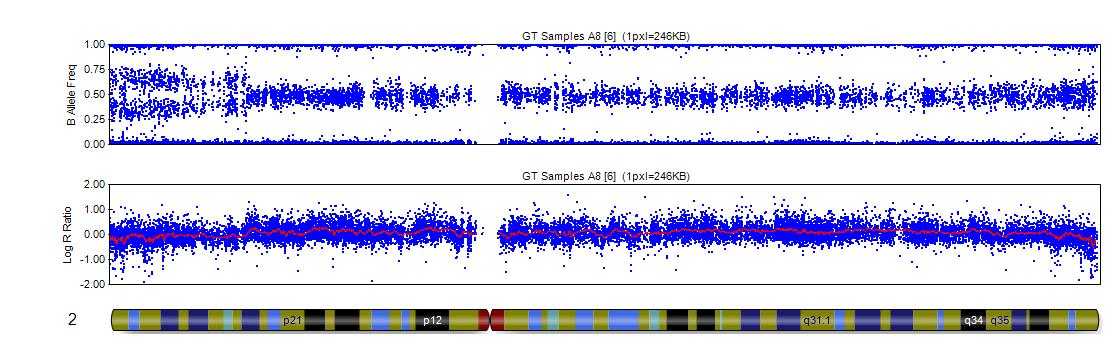


**B7: 46, XX**


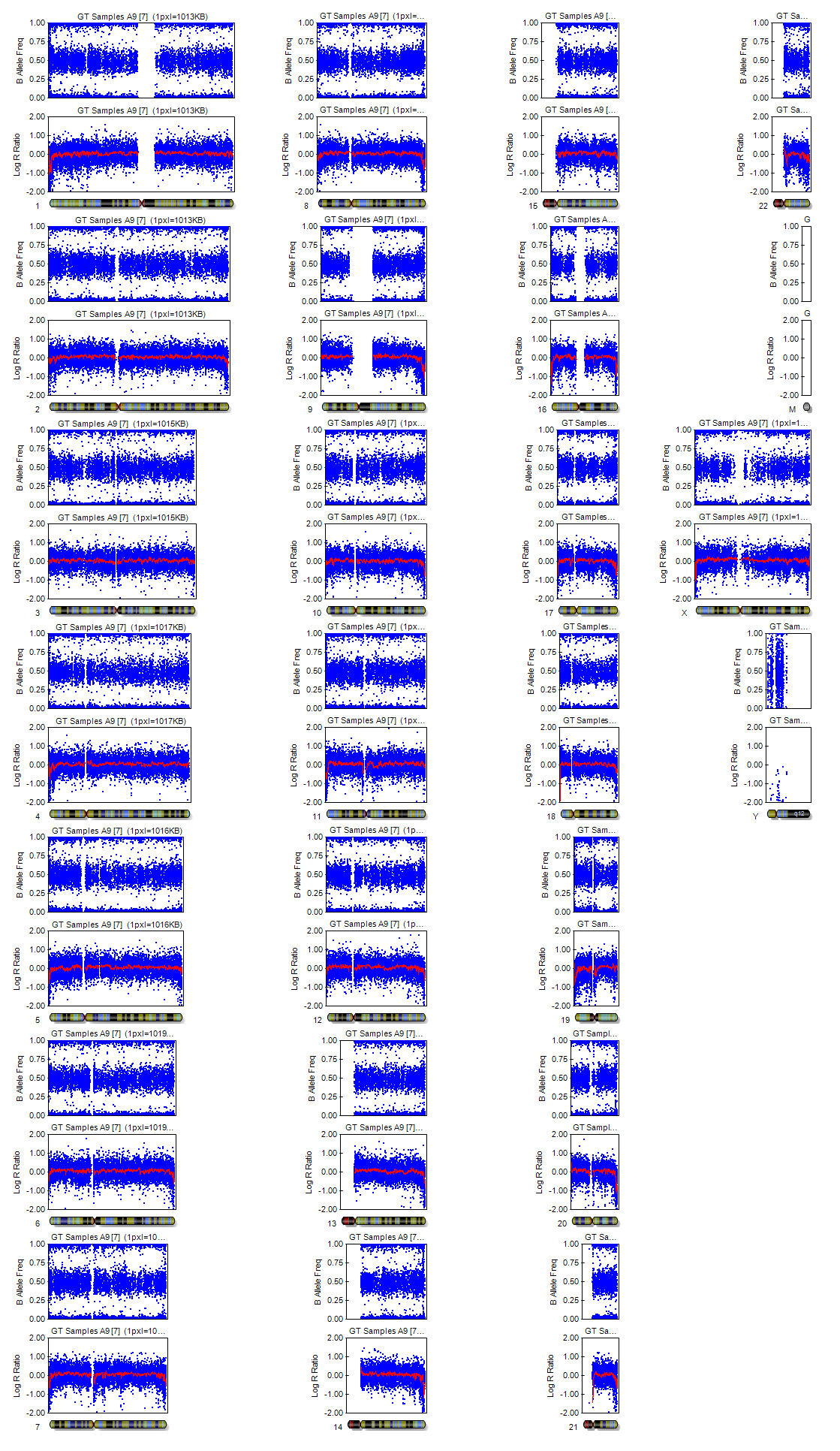


**Supplementary Figure 1. Chromosomal aneuploidy of blastocysts derived from rescue activation of calcium ionophore (A23187)**

SNP karyotype analysis was performed on 7 blastocysts (B1-B7) obtained after rescue activation of unfertilized oocytes after ICSI. The results showed that the karyotypes of the 3 blastocysts were normal, namely B3 (46, XY), B5 (46, XX) and B7 (46, XX). The karyotype of 4 blastocysts is abnormal: the karyotype of blastocyst B1 is 45, XX, -13, multiple uniparental disomy (UPD), the blastocyst has one missing chromosome 13, and the rest of the chromosomes are UPD, multiple UPD may be caused by embryonic genome self-repair or parthenogenesis; the karyotype of blastocyst B2 is dup (1) (p32.244), +2, +3, +4, +5, +6, +7, +8, +9, + 10, +11, +12, +15, +16, +17, +18, +19, +20, upd (21) (q11.2-q21.3), +22, XXY, and this chromosomal abnormality is equivalent to triploid; the karyotype of blastocyst B4 is 45, XX, -14, dup (10) (pter-q22.2), del (10) (q22.3-q26.3), and this blastocyst is missing chromosome 14, also with pter-q22.2 segmental duplication and q22.3-q26.3 segmental deletion on chromosome 10; the karyotype of blastocyst B6 is 46, XX, dup(2)(pter-p23.1), and this blastocyst has a segmental duplication of pter-p23.1 on chromosome 2.
